# Screening of potential antioxidant bioactive Q-markers of paeoniae radix rubra based on an integrated multimodal strategy

**DOI:** 10.3389/fphar.2024.1447959

**Published:** 2024-08-15

**Authors:** Hengli Li, Yu Zhao, Jiaqi Wang, Caiwang Peng, Keyan Tang, Mu Sun, Yantao Yang, Qingping Liu, Fang Liu

**Affiliations:** ^1^ School of Pharmacy, Hunan University of Chinese Medicine, Changsha, Hunan, China; ^2^ Center for standardization and functional engineering of traditional Chinese medicine in Hunan province, Changsha, Hunan, China; ^3^ Key Laboratory of Modern Research of TCM, Education Department of Hunan Province, Changsha, Hunan, China; ^4^ School of Informatics, Hunan University of Chinese Medicine, Changsha, Hunan, China

**Keywords:** quality markers (Q-markers), paeoniae radix rubra (PRR), antioxidant, spectrum-efficacy correlation, network pharmacology, molecular docking

## Abstract

**Background:**

Paeoniae Radix Rubra (PRR) has been used widely to promote blood circulation and eliminate blood stasis in China clinical practice owing to its extensive pharmacological effects. However, the “quality markers” (Q-markers) of the antioxidant effects remains unknown.

**Object:**

To explore the Q-markers of antioxidant activity based on multiple strategies, which would provide reference for the quality evaluation of PRR based on specific pharmacodynamic-oriented.

**Methods:**

Firstly, the “fingerprint” profiles of 15 batches of PRR were acquired and identified by ultrahigh performance liquid chromatography-quadrupole time-of-flight tandem mass spectrometry (UHPLC-Q-TOF MS/MS) and the common peaks extracted. Meanwhile, the MTT assay was used to evaluate the effect of 15 batches of PRR on H_2_O_2_-induced oxidative stress in HT-22 cells. The antioxidant activity of PRR was investigated simultaneously by superoxide dismutase (SOD), glutathione (GSH), and malondialdehyde (MDA) commercial kits. The relationship between common peaks and antioxidant indexes were constructed by grey relational analysis (GRA) and partial least squares-discriminant analysis (PLS-DA) for the identification of preselected Q-markers. Secondly, experimental verification was conducted to investigate the protective effect of the preliminary components on HT-22 cells undergoing oxidative stress. Finally, for the further validation of effectiveness of antioxidant Q-markers, network pharmacology was applied to explore potential targets, and the molecular docking technology was used to value the binding ability of the potential active components of PRR to the antioxidant targets.

**Results:**

Thirty-seven common peaks from 15 batches of PRR were identified qualitatively by UHPLC-Q-TOF MS/MS. The MTT assay showed that PRR could reduce the oxidative damage induced by H_2_O_2_ upon HT-22 cells according to the index of MDA, SOD and GSH. Eight potential antioxidant components were screened by spectrum-effect correlation analysis: paeoniflorin, galloylpaeoniflorin, albiflorin, 1,2,3,4,6-o-pentagalloylglucose, benzoylpaeoniflorin, pinocembrin, oleanic acid, and isorhamnetin-3-o-nehesperidine. Each of these preliminary components showed significant protections on cellular oxidative stress (*P <* 0.05). Interleukin-6 (IL-6), protein kinase B (AKT1), and tumor necrosis factor (TNF) were predicted to be the major potential targets of PRR, and the good binding ability were presented between the potential active components of PRR and each target as a whole.

**Conclusion:**

Eight components were identified as the antioxidant Q-markers of PRR based on an integrated multimodal strategy.

## 1 Introduction

Paeoniae Radix Rubra (PRR) is the root of *Paeonia lactiflora Pall* or *Paeonia veitchii Lynch*. It was first recorded in *ShenMungHerbal*. PRR has been used widely to promote blood circulation and eliminate blood stasis in clinical practice in China because it has anti-thrombotic (Ye et al., 2016), anti-ischemic (Zhao et al., 2022), anti-inflammatory (Zhang and Wei, 2020), and antioxidant properties (Li S. et al., 2018). Our previous studies have shown that PRR exerts neuroprotective effects against cerebral ischemia by inhibiting neuronal ferroptosis via antioxidant pathways. (Zhao et al., 2023). Deficiency in anti-oxidative function has been postulated to be associated with various diseases. Excessive accumulation of free radicals is a crucial process in the pathogenesis of ischemic diseases, aging, and cancer. PRR has been shown to counteract the accumulation of free radicals, which is associated with the effect of stasis-dispersing probably (Xu et al., 2022). PRR exerts therapeutic effects on liver damage (Liu T. et al., 2022), atherosclerosis (Li C. et al., 2018), cognitive dysfunction (Liu et al., 2020), and pulmonary fibrosis (Liu et al., 2017) by inhibiting excessive cellular oxidation. This phenomenon indicates that screening for the active components in PRR responsible for its antioxidant effects is a rational approach. In addition, the quality of PRR affects its efficacy directly. The strength of an antioxidant can reflect intuitively the specific bioactive components. However, the quantity and content of chemical components are different if PRR is extracted from different parts of the host plant or if different preparation technologies are adopted. For instance, Tong and colleagues (Tong N.-N. et al., 2021) investigated the chemical composition and antioxidant activity of stems and leaves separately, as well as 10 parts, of PRR. They found that different parts of PRR had different levels of antioxidant activity. However, studies on the PRR components that are efficacious against different diseases are scarce. Therefore, it is particularly essential to establish a direct correlation among components, effects, and diseases, which would serve as a scientific approach to evaluate the quality of traditional Chinese medicine (TCM).

“Chemical fingerprinting” and qualitative analysis of multiple chemical components have been shown to be powerful methods to judge the efficacy and safety of TCM (Noviana et al., 2022). High-performance liquid chromatography (HPLC) and ultrahigh-pressure liquid chromatography/tandem mass spectrometry (UPLC-MS/MS) have been used widely to identify and evaluate the quality of complex multicomponent TCM. However, these methods characterize (qualitatively and quantitatively) the chemical components contained in a TCM without detailing the correlations between specific effects and chemical components. Therefore, clearly identifying the pharmacological-material basis responsible for specific effects is challenging. Also, there are limitations in evaluating the quality of a TCM from a clinical medication.

As a promising concept, “quality markers” (Q-markers) are derived from the “nature–effect–component” theory proposed by Liu et al. (2016). This theory closely links the relationship among the material basis, efficacy, and Q-markers of a TCM. Analyses of the spectrum–effect can reflect the correlation between chemical components and pharmacological activities (He et al., 2023). This is a commonly used and convenient concept for screening Q-markers. It is significant to further identify the potential pharmacological material bases and provide feasible solutions for evaluating the quality standards of TCM, also clarifying the material basis.

**FIGURE 1:**
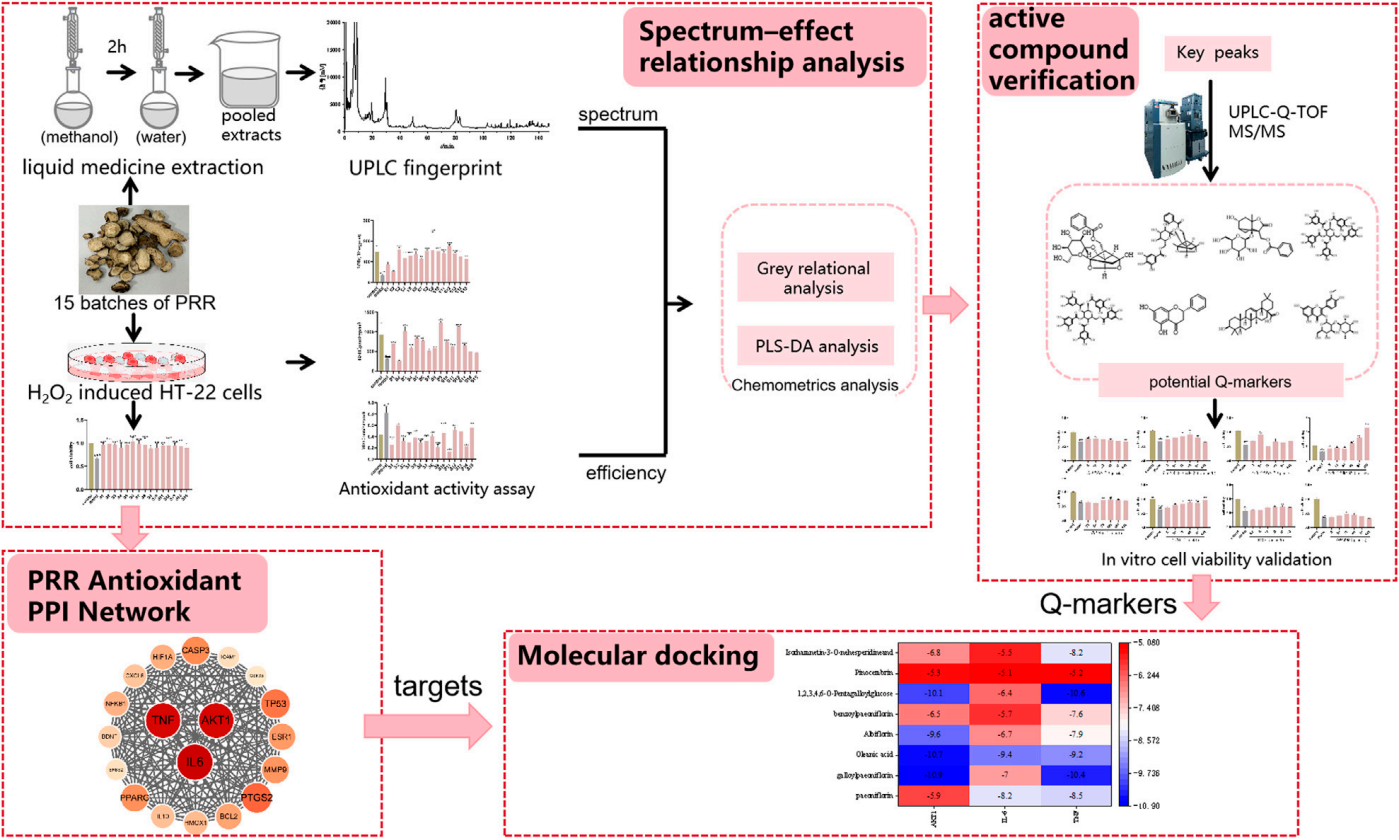
Graphical abstract.

We integrated multiple strategies based on Q-marker screening. Initially, we characterized the chemical components and evaluated the antioxidant activity of PRR. Subsequently, spectrum–effect relationships were established using various chemometric methods to preliminarily screen out the bioactive components associated with the antioxidant activity of PRR. *In vitro* experiments were conducted to evaluate the antioxidant effect of each bioactive component using mouse hippocampal neuronal (HT-22) cells. Network pharmacology was employed to explore the biological targets of antioxidant activity. Molecular docking was conducted to confirm the relationship between components and targets. Ultimately, the Q-markers for the antioxidant effects of PRR were identified. This strategy for the screening of Q-markers could provide a reference for efficacy-oriented quality evaluation of PRR. The graphical abstract is shown in Figure 1.

## 2 Materials and methods

### 2.1 Materials

Fifteen batches of *P. lactiflora Pall* or *P. veitchii Lynch*. Herbs were identified by Professor Zhou Xiao Jiang (Hunan University of Chinese Medicine, Changsha, China) according to the 2020 edition of *Chinese Pharmacopoeia* (Chinese Pharmacopoeia Commission, 2020). The batch numbers for sample production corresponding to numbers are shown in Table 1. Samples were stored in the Center for Standardization and Functional Engineering of Traditional Chinese Medicine in Hunan Province (Changsha, China).

**TABLE 1:** Information of 15 batches of PRR samples.

Number	Locality of growth	Origin	Number	Locality of growth	Origin
S1	Xinfan County, Sichuan	Paeonia veitchii Lynch	S9	Jinchuan County, Sichuan	Paeonia veitchii Lynch
S2	Zhongjiang County, Sichuan	Paeonia veitchii Lynch	S10	Xichang City, Sichuan	Paeonia veitchii Lynch
S3	Beichuan Qiang Autonomous County, Sichuan	Paeonia veitchii Lynch	S11	Heishui County, Sichuan	Paeonia veitchii Lynch
S4	Rang tang County, Sichuan	Paeonia veitchii Lynch	S12	Rangtang County, Sichuan	Paeonia veitchii Lynch
S5	Jiuzhaigou Sichuan	Paeonia veitchii Lynch	S13	Unknown	Paeonia lactiflora Pall
S6	Quwo County, Shanxi	Paeonia lactiflora Pall	S14	Unknown	Paeonia lactiflora Pall
S7	Zhongjiang County, Sichuan	Paeonia veitchii Lynch	S15	Unknown	Paeonia veitchii Lynch
S8	Heishui County, Sichuan	Paeonia veitchii Lynch			

Eight types of reference standards were purchased from Chengdu Aifa Biotechnology (Chengdu, China): paeoniflorin, galloylpaeoniflorin, albiflorin, 1,2,3,4,6-o-pentagalloylglucose, benzoylpaeoniflorin, pinocembrin, oleanic acid, and isorhamnetin 3-o-neohesperidoside. The purity of all reference standards were higher than 98%. The H_2_O_2_ solution was obtained from Sigma (Shanghai, China). Chromatography-grade acetonitrile and methanol were purchased from Merck (Darmstadt, Germany). Deionized water was purified by the Milli-Q™ system (Millipore, Bedford, MA, United States). Superoxide dismutase (SOD), glutathione (GSH), and malondialdehyde (MDA) assay kits (S0021) with batch numbers of E-BC-K020-M, E-BC-K030-M, and E-BC-K028-M, respectively, were from Elabscience Biotechnology (Wuhan, China).

### 2.2 Qualitative analyses of PRR

#### 2.2.1 Preparation of the sample solution of PRR

Fifteen batches of PRR were extracted ultrasonically thrice with 70% ethanol. The filtrate was collected after suction filtration to remove impurities. Ethanol was recovered by vacuum distillation with a rotary evaporator, and then dried *in vacuo* to obtain the dry extract. Each gram was equivalent to 4 g of crude drug. Methanol (100%) was used to dissolve PRR extracts. After ultrasonic treatment for 30 min and passage through a 0.22 μm filter membrane, solution samples for UHPLC were obtained. DMEM was added to dissolve the PRR extracts at appropriate concentrations for cell experiments.

#### 2.2.2 Ultrahigh performance liquid chromatography-quadrupole time-of-flight tandem mass spectrometry (UHPLC-Q-TOF MS/MS)

Chromatographic separation was undertaken using an Ultimate UPLC XB-C18 column (1.8 µm, 2.1 × 100 mm). The mobile phase consisted of pure water with 0.1% formic acid solution (A) and acetonitrile (B) at a flow rate of 0.3 mL/min. The gradient program was: (A:B, *v/v*): 0–26.67 min, 10%–15% (B); 26.67–93.35 min, 15%–22% (B); 93.35–120.21 min, 22%–55% (B); 120.21–146.92 min, 45%–50% (B). The volume of sample for injection was 3 μL.

The high-resolution quadrupole time-of-flight mass spectrometer equipped with an ESI source was used as the detector. Positive and negative ionization modes were used at the following MS parameters: first cone voltage, −80 V to −80 V; first collision voltage, −10 V to −10 V; second cone voltage, −80 V to −80 V; second collision voltage, −50 V to −20 V. Data analysis was done using SCIEX OS 1.2 software.

### 2.3 *In vitro* cell experiments

#### 2.3.1 Cell culture and model of oxidative stress

HT-22 cells (STR identification confirmed, CL-0697) were purchased from Hunan Kangheyi Biotechnology (Hunan, China) and cultured in DMEM supplemented with 10% fetal bovine serum and 1% antibiotics at 37°C in an incubator in an atmosphere of 5% CO_2_. After reaching 80% confluence, cells were seeded into 96-well plates at 10^3^–10^4^ per well. Cells were allowed to react with H_2_O_2_ (0.9 μM) for 2 h to create a model of oxidative stress according to a previous study (Supplementary Figure S1) to permit 60%–70% of cells to become injured.

#### 2.3.2 Cell activity

After completing cell culture, cells were pretreated for 2 h with PRR (120 μg⋅mL^−1^) or vehicle (DMEM) as the drug-intervention group and control group, respectively. Then, H_2_O_2_ (0.9 μM) was added to the model group and drug-intervention group for 2 h. Cell viability was quantified using the MTT assay. Data were normalized to vehicle-treated cells (100%) and five replicates were set up in parallel in each group.

Cell viability based on optical density (OD) was evaluated using the MTT assay as described previously using this equation:

$$\text{Cell viability}=\left(\text{OD }-\;{\text{OD}}_{\text{blank}}\right)/\left({\text{OD}}_{\text{control}}\;-\;{\text{OD}}_{\text{blank}}\right)$$

#### 2.3.3 Antioxidant activity

Cells were cultured using the same method as described in Section 2.3.1. SOD activity and levels of GSH and MDA were measured according to instructions set on the respective assay kits.

### 2.4 Spectrum–effect relationship

The spectrum–effect relationship between the UHPLC fingerprints and the SOD, GSH and MDA of PRR extracts were analyzed by the grey relational analysis (GRA) and partial least squares-discriminant analysis (PLS-DA). The indexes of antioxidant effect were used as the reference sequence, and the common characteristic peaks of the fingerprint profile (S1–S15) were listed as the comparative sequence. The contribution of each common peak to the efficacy was determined by comparing the gray correlation between the comparative sequence and the reference sequence. SPSS AU (https://spssau-com/) and SIMCA was undertaken by Umetrics (Stockholm, Sweden) software were used to analyze the gray correlation degree and partial least squares method respectively.

### 2.5 Verification of the antioxidant effect of eight monomer components *in vitro*

Oxidative stress model was constructed on HT-22 cells by H_2_O_2_. To investigate the effect of each preliminary compound on the survival rate of oxidative stress cell model, HT-22 cells were divided into the three groups: control (normal conditions), oxidative-stress model (treated with H_2_O_2_ for 2 h at the concentration of 0.9 μM) (Supplementary Figure S1), and each monomer component pretreated groups. According to the literature analysis, cells were respectively treated with paeoniflorin (5, 10, 20, 40, 80, 160 μM) (Liu Y.-F. et al., 2022), galloylpaeoniflorin (5, 10, 20, 40, 80, 160 μM) (Wen et al., 2018), oleanolic acid (5, 10, 20, 40, 80, 160 μM) (Jiang et al., 2020), albiflorin (25, 50, 75, 100, 125, 150 μM) (Xue et al., 2023), benzoylpaeoniflorin (2.5, 5, 10, 20, 40, 80 μM) (Kim et al., 2022), 1,2,3,4,6-o-pentagalloylglucose (5, 10, 20, 40, 80, 160 μM) (Tong J. et al., 2021), pinocembrin (2.5, 5, 10, 20, 40, 80 μM) (Wang T. et al., 2022), or isorhamnetin 3-o-neohesperidoside (5, 10, 20, 40, 80, 160 μM).

### 2.6 Network pharmacology

Target information of the active compounds in PRR was collected from PubChem (https://pubchem.ncbi.nlm.nih.gov/) and Traditional Chinese Medicine Systems Pharmacology Database and Analysis Platform (TCMSP; http://tcmspw.com/) databases. All PRR targets collected were combined and de-duplicated, then corrected on the Universal Protein database (https://www.uniprot.org/), and converted into the official gene symbol of *Homo sapiens*. Oxidation-related targets were obtained from GeneCards (www.genecards.org/), Online Mendelian Inheritance in Man (OMIM; www.omim.org/), TherapeuticTargetDatabase (www.idrblab.net/), and DrugBank (https://go.drugbank.com/). They were obtained by using “oxidation” as the keyword and removing duplicate and false-positive genes, and the intersection target genes of the two (PRR–oxidation targets) were collected as the potential targets of PRR for antioxidants. Then, we imported the PRR–oxidation targets into the Search Tool for the Retrieval of Interacting Genes/Proteins database (https://cn.string-db.org/) setting the condition as “human” (*H. sapiens*). Selected data with a combined score ≥0.9 was imported into Cytoscape 3.9.1 (https://cytoscape.org/), and we constructed a protein–protein interaction (PPI) network. The degree values of the topological parameter in the network were used to identify the core target genes in the PPI network.

### 2.7 Molecular docking

The 3D structures of proteins interleukin (IL)-6, protein kinase B (AKT1), and tumor necrosis factor (TNF) were obtained from Structural Bioinformatics Protein Data Bank (www.rcsb.org/). AutoDock Vina (Trott and Olson 2010) was used for molecular docking of active components with protein IL-6, TNF, AKT1. During the docking process, the protein structures were converted to a PDBQT file, which contained all the polar residues with hydrogen. The bioactive components were also converted to a PDBQT file and all the bonds were set to be rotatable. The receptor was set to be fixed while the ligand was allowed to have a certain flexibility, and this flexible docking simulation was carried out using the Lamarcian genetic algorithm. A search of possible conformations of the ligand was performed within the confines of the grid box and the conformation with the lowest binding free energy was finally identified as the best probable conformation. The models of the complex were analyzed using Discovery Studio (BIOVIA Dassault Systèmes 2017). Surflex-Dock scores (total scores) were expressed in kcal/mol units to represent binding affinities (Feinstein and Brylinski, 2015).

### 2.8 Statistical analyses

SPSSAU and SIMCA were used for GRA and PLS-DA. Data from cell experiments were analyzed using Prism 9.0 (GraphPad, La Jolla, CA, United States). The Student’s *t*-test and one-way analysis of variance were applied for statistical analyses.

## 3 Results

### 3.1 Chemical fingerprinting and phytochemical analysis of PRR

The total ion chromatograms of the PRR extract acquired by UPLC-Q-TOF MS/MS in positive and negative ion modes are shown in Supplementary Figure S2. The retention time, accurate quality database, and mass-spectrum library of PRR were established by SCIEXOS1.2 software. The resultant 43 compounds were identified qualitatively (Table 2) by comparison of information of primary MS and secondary MS, as well as information in the literature.

**TABLE 2:** Results of UPLC-Q-TOF-MS identification of the components of PRR.

Number	Molecule name	T_R_/min	Formula	Molecular weight (m/z)	Error (ppm)	MS^2^	CAS
1	L-Malic acid ([M-H])	1.02	C_4_H_6_O_5_	133.0138	2.7	115.0036/72.0138/59.0138	97-67-6
	5-hydroxyfuran-2-carbaldehyde ([M-H])	1.02	C_5_H_4_O_3_	126.0341	3.8	109.0284/97.0284/81.033469.0334/53.0021	67-47-0
2	Citric acid ([M-H])	1.435	C_6_H_8_O_7_	191.0191	5.1	111.0087/85.0295/67.0189/57.0345	77-92-9
	Gallic acid ([M-H])	1.435	C_7_H_6_O_5_	169.0140	4.1	125.0244/107.0138/97.0295/69.0345/67.0189	149-91-7
3	protocatechuic acid ([M-H])	2.419	C_7_H_6_O_4_	153.0192	−0.9	109.0295/91.0189/81.0345/65.0032/53.0396	99-50-3
	3,4-Dihydroxy-5-methoxybenzoic acid ([M-H])	2.419	C_8_H_8_O_5_	183.0295	−2.2	168.0064/124.0166/106.0060	3,934-84-7
4	Methyl gallate ([M-H])	3.288	C_8_H_8_O_5_	183.0293	0.5	168.0064/124.0166/78.0111	99-24-1
	2,5-Dihydroxybenzoic acid ([M-H])	3.288	C_7_H_6_O_4_	153.0189	6.2	108.0127	490-79-9
5	Ethyl gallate ([M-H])	7.888	C_9_H_10_O_5_	197.0451	−2.3	169.0139/125.0242/124.0159/106.0056/78.0104	831-61-8
6	1,3,4,6-Tetra-O-galloylglucose ([M-H])	10.596	C_34_H_29_O_22_	787.0999	1.1	635.0889/617.0784/483.0780,466.0752/447.0568/313.0565/295.0459/169.0142	26,922-99-6
7	1,3,6-Tri-O-galloylglucose ([M-H])	11.785	C_27_H_24_O_18_	635.0889	0.6	483.0780/465.0674/423.0568/313.0565/295.0459/169.0142/125.0244	18,483-17-5
8	Albiflorin ([M-H])	13.823	C_23_H_28_O_11_	525.1590	0.2	449.1441/327.1076/165.0551/121.0288/77.0393	39,011-90-0
	Paeoniflorin ([M-H])	13.823	C_23_H_28_O_11_	479.1976	0.8	327.0708/169.0140/121.0286/77.0391	23,180-57-6
	6′-O-Galloyl paeoniflorin ([M-H])	13.823	C_30_H_32_O_15_	631.1638	2.8	613.1544/509.1294/491.1182/479.1194/313.0552/271.0446/169.0132	122,965-41-7
9	1,3,4,6-Tetragalloylglucose ([M-H])	14.78	C_34_H_29_O_22_	787.0999	1.1	635.0889/617.0784/483.0780,466.0752/447.0568/313.0565/295.0459/169.0142	26,922-99-6
10	6′-O-Galloyl paeoniflorin ([M-H])	19.627	C_30_H_32_O_15_	631.1638	2.8	613.1544/509.1294/491.1182/479.1194/313.0552/271.0446/169.0132	122,965-41-7
11	1,2,3,4,6-O-Pentagalloylglucose ([M-H])	28.929	C_41_H_32_O_26_	939.1059	3.2	787.0974/769.0855/617.0756/447.0553/169.0132	14,937-32-7
12	Lactiflorin ([M-H])	30.649	C_23_H_26_O_10_	507.1004	1.7	339.1091/177.0545/121.0289	1,361,049-59-3
13	Lactiflorin ([M-H])	37.172	C_23_H_26_O_10_	507.1004	1.7	339.1091/177.0545/121.0289	1,361,049-59-3
14	Kaempferol-3-O-(6″-galloyl)-β-glucopyranoside ([M-H])	38.618	C_28_H_24_O_15_	599.1100	8.4	169.0142/123.0088	56,317-05-6
15	1,3,4,6-Tetragalloylglucose ([M-H])	46.211	C_34_H_29_O_22_	787.0999	0.6	635.0904/617.0720/483.1880/465.0671/295.0667/169.0144	26,922-99-6
16	(Z)-(1S,5R)-β-pinen-10-yl-β-v icianoside ([M-H])	49.198	C₂₁H₃₄O₁₀	491.2114	—	445.2066/293.0867/149.0448/89.0238	—
17	Benzoylpaeoniflorin ([M-H])	80.499	C_30_H_32_O_12_	629.1162	1.8	553.1705/431.1341/165.0551/121.0287/77.0393	38,642-49-8
18	Benzoylpaeoniflorin ([M-H])	93.099	C_30_H_32_O_13_	629.1162	1.8	553.1705/431.1341/165.0551/121.0287/77.0393	38,642-49-8
19	Asiatic acid ([M-H])	103.139	C_30_H_48_O_5_	487.3467	7.8	345.2097/317.2117/96.9594	464-92-6
20	Rubusoside ([M-H])	103.785	C_32_H_50_O_13_	641.3178	1.7	479.2638	64,849-39-4
21	Pinocembrin ([M-H])	108.611	C_15_H_12_O_4_	255.0528	1.0	213.0577/183.0645/171.0451/145.0670	480-39-7
22	Sedanolide ([M+H])	109.531	C_12_H_18_O_2_	195.1741	1.7	107.0855/99.0440/79.0542/69.0698/67.0542	6,415-59-4
23	FragransinA2 ([M+H])	110.782	C_20_H_24_O_5_	345.1690	5.2	203.1056/137.0592	112,652-46-7
24	4-Hydroxybenzoic acid ([M-H])	111.47	C_7_H_6_O_3_	137.0238	−4.5	93.0351	99-96-7
25	Regaloside A ([M+H])	112.084	C_18_H_24_O_10_	423.2890	0.7	173.0442/101.0237	114,420-66-5
26	Diammonium glycyrrhizinate ([M-H])	113.036	C_42_H_65_NO_16_	821.4391	0.6	351.0569/	79,165-06-3
27	Linolelaidic acid ([M+H])	117.885	C_18_H_30_O_2_	279.2317	1.5	57.0699	506-21-8
28	Acacetin ([M-H])	119.536	C_16_H_12_O_5_	285.1470	1.7	139.0393/55.0542	480-44-4
29	Emodin ([M+H])	120.155	C_15_H_10_O_5_	271.1319	2.1	105.0335	518-82-1
30	Neohesperidin ([M-H])	120.652	C_28_H_34_O_15_	609.2914	0.6	295.0459/166.9986/125.0244/123.0088	13,241-33-3
	Glycyrrhetinic acid ([M-H])	120.652	C_30_H_46_O_4_	469.2933	6.0	151.0401/135.0452/	471-53-4
31	Oleanic acid ([M-H])	124.219	C_30_H_48_O_3_	455.3123	−0.8		508-02-1
	Isorhamnetin 3-O-neohesperidoside ([M-H])	124.219	C_28_H_32_O_16_	623.2799	7.1	169.0142/152.9982/124.0165	55,033-90-4
32	gingerglycolipid B ([M-H])	131.2	C_33_H_58_O_14_	723.4175	1.1	677.3754/415.1457/397.1351/287.0772/253.0929/235.0823/161.0455	88,168-90-5
33	Ursolic Acid ([M+H])	132.981	C_30_H_48_O_3_	457.3577	1.4	145.1012/131.0855	77-52-1
34	Hederagenin ([M-H])	133.696	C_30_H_48_O_4_	471.3480	0	471.3480	465-99-6
35	N-Benzyloleamide2 ([M+H])	134.487	C_26_H_43_NO_2_	372.3459	0.9	88.0756/57.0698/55.0542/	883,715-21-7
36	Cinnamic acid ([M+H])	139.449	C_9_H_8_O_2_	149.0231	−5.9	121.0284	621-82-9
37	Scoparone ([M+H])	142.106	C_11_H_10_O_4_	207.0304	−2.1	105.0699	120-08-1

**FIGURE 2:**
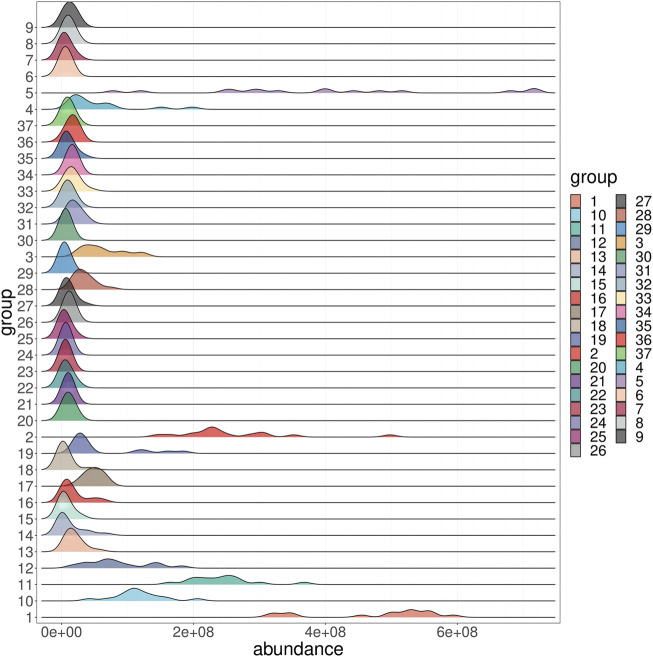
The distribution map of 37 common components in 15 batches of PRR.

In order to more intuitively observe the content distribution of components in PRR and distinguish between principal and differential components, a heat map analysis was performed on 15 batches of PRR. The content distribution was expressed in the degree of color depth, and gray indicated that the peak did not exist in the batch of PRR. The results showed that the color differences of peaks 1, 2, 3, 4, 5, 10, 11, 12, and 18 in each batch of PRR were large, indicating that the content distribution was not uniform. The disappearance of peaks 16, 20, 29, 30, 33, 42, 44, and 45 in some batches of PRR can be defined as differential components (Supplementary Figure S3). In order to further understand the distribution of 37 common peaks in each batch of PRR, a probability distribution analysis was carried out (Figure 2). The peaks 1, 2, 3, 4, 5, 10, 11, and 12 were unevenly distributed, and the peaks 14, 16, 17, 19, and 28 were distributed to the right. The remaining peaks were approximately normally distributed, indicating that most of the components were uniformly and stably distributed in the PRR. The results were consistent with the heat map analysis.

### 3.2 Protective effects of PRR on oxidative stress in cells

**FIGURE 3:**
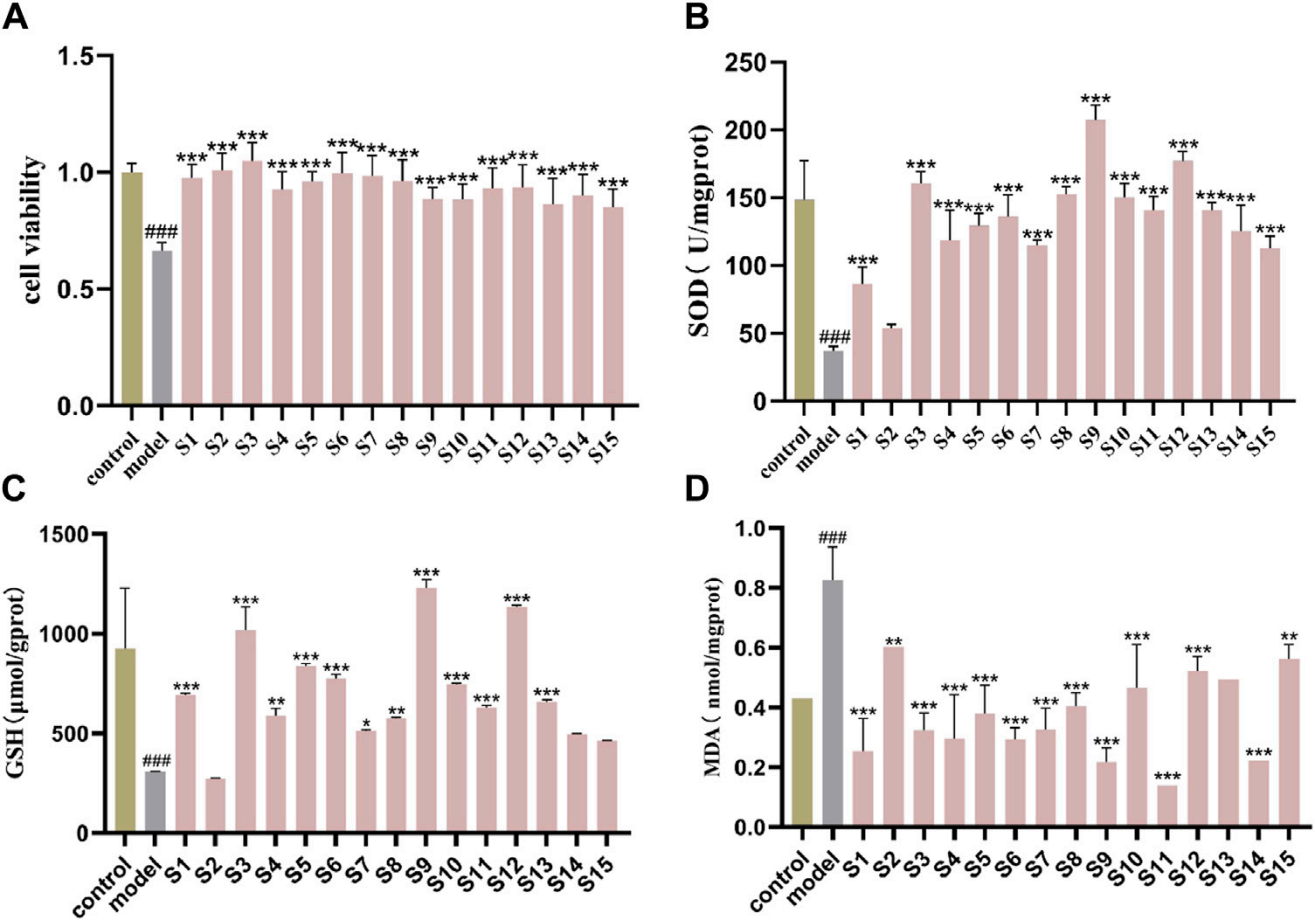
Protective effect of 15 batches of PRR on oxidant stress cells model **(A)** cell viability; **(B)** SOD activity; **(C)** GSH content; **(D)** MDA content.mean ± SD, n = 3 ###*P* < 0.001 vs. control group; *P < 0.05, **P < 0.01, ***P < 0.001 vs. model group.

The protective effects of 15 batches of PRR extracts on cell survival are shown in Figure 3A. The percentage of cells that survived in the H_2_O_2_-induced model group decreased to 60%–70%, and this difference was significant compared with the normal control group (*P < 0.001*). Each PRR extracts displayed favorable proliferation of HT-22 cells undergoing oxidative stress injure, which indicated the potential antioxidant bioactive of PRR. Additional results on the antioxidant bioactive of PRR are shown in Figures 3B–D. The activity level of GSH and SOD in the model group were reduced significantly compared with those in the control group (*P < 0.001*), but MDA content was increased significantly (*P < 0.001*), which demonstrated that a cellular model of oxidative stress had been created. The PRR extracts could prompt the three antioxidant index to a normal level, except for the second batch. Though the differences were existed among in the different batches of PRR, the all displayed a significant difference compared with model group, even up to the viability of normal control group. The comprehensive results showed that PRR exerts a protection against oxidative stress cells induced by H_2_O_2_.

### 3.3 Spectrum−effect relationship analysis by GRA and PLS-DA

**FIGURE 4:**
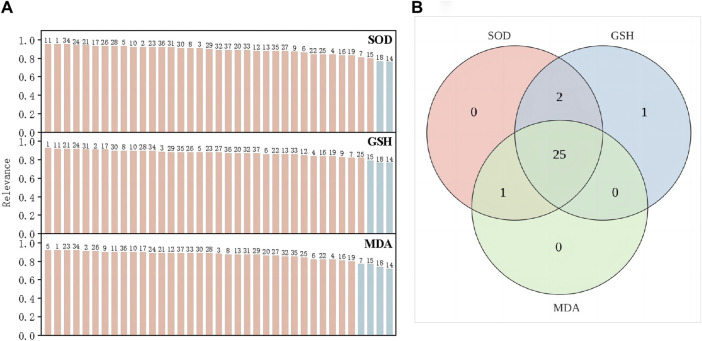
SOD, GSH and MDA gray correlation coefficient greater than 0.85 **(A)**. the intersection peaks with gray correlation coefficients of the three greater than 0.85 **(B)**.

The degree of correlation obtained according to GRA is shown in Supplementary Tables S1–S3. The correlation coefficients of the antioxidant activities of SOD, GSH, and MDA were >0.6 (Table 3), indicating that SOD, GSH, and MDA were correlated with the common peaks of PRR. A correlation coefficient >0.85 represented a strong correlation. The gray correlation coefficients of the three antioxidant activities were sorted in order. The results are shown in Figure 4A. Twenty-five peaks with a correlation coefficient >0.85 (Figure 4B) (1, 2, 3, 5, 8, 10, 11, 12, 13, 17, 20, 21, 23, 24, 26, 27, 28, 29, 30, 31, 32, 33, 34, 36, 37) were obtained.

**TABLE 3:** Correlation sequence between 37 common peaks of PRR and antioxidant index.

Association number	SOD		GSH		MDA	
	Peak number	Correlation coefficient	Peak number	Correlation coefficient	Peak number	Correlation coefficient
1	11	0.95	1	0.93	5	0.92
2	1	0.95	11	0.92	1	0.92
3	34	0.95	21	0.92	23	0.92
4	24	0.94	24	0.92	34	0.92
5	21	0.94	31	0.91	2	0.91
6	17	0.93	2	0.91	26	0.91
7	26	0.93	17	0.91	9	0.90
8	28	0.93	30	0.90	11	0.90
9	5	0.93	8	0.90	36	0.90
10	10	0.92	10	0.90	10	0.90
11	2	0.92	28	0.90	17	0.90
12	23	0.92	34	0.90	24	0.89
13	36	0.92	3	0.89	21	0.89
14	31	0.92	29	0.88	12	0.89
15	30	0.91	35	0.88	37	0.89
16	8	0.91	26	0.88	33	0.89
17	3	0.91	5	0.88	30	0.89
18	29	0.90	23	0.88	28	0.89
19	32	0.89	27	0.88	3	0.88
20	37	0.89	36	0.87	8	0.87
21	20	0.89	20	0.87	13	0.87
22	33	0.89	32	0.87	31	0.87
23	12	0.88	37	0.87	29	0.87
24	13	0.88	6	0.86	20	0.86
25	35	0.88	22	0.86	27	0.86
26	27	0.88	13	0.86	32	0.85
27	9	0.87	33	0.86	35	0.85
28	6	0.86	12	0.85	25	0.84
29	22	0.84	4	0.84	6	0.82
30	25	0.84	16	0.84	22	0.82
31	4	0.84	19	0.84	4	0.82
32	16	0.83	9	0.83	16	0.81
33	19	0.83	7	0.82	19	0.80
34	7	0.81	25	0.82	7	0.77
35	15	0.80	15	0.79	15	0.77
36	18	0.77	18	0.77	18	0.74
37	14	0.76	14	0.77	14	0.72

**FIGURE 5:**
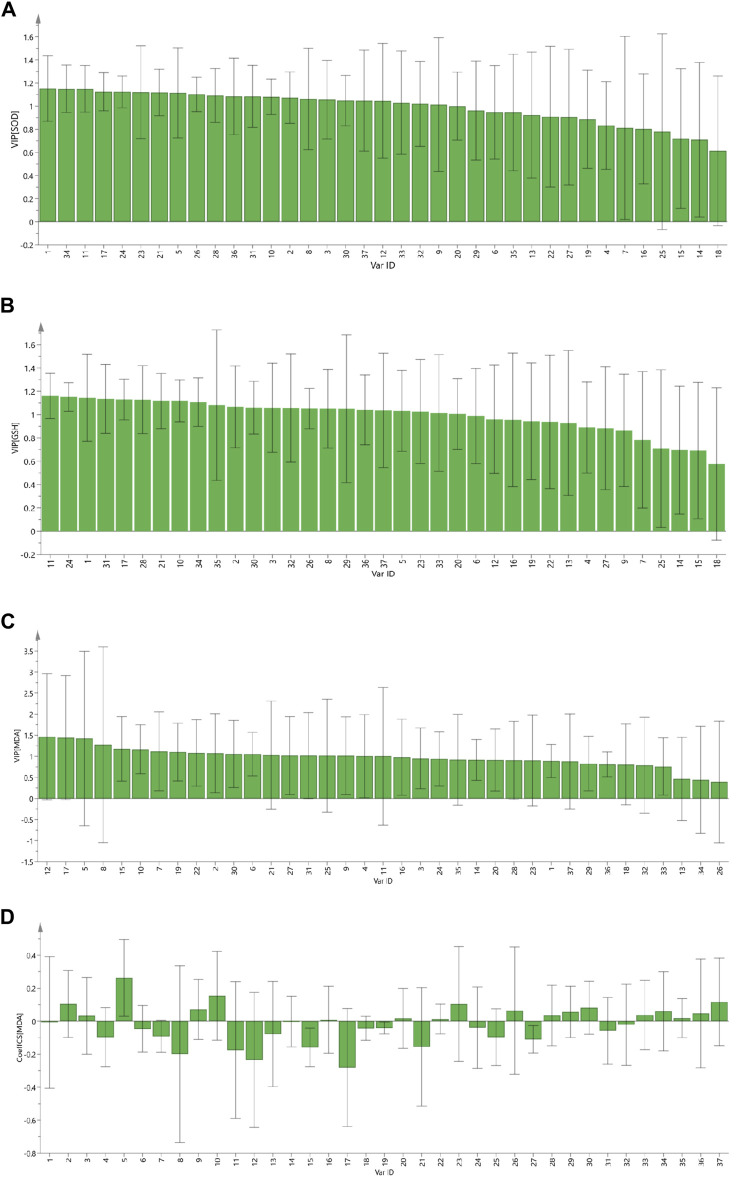
The VIP contribution of 37 common peaks to the antioxidant activity of PRR. SOD **(A)**; GSH **(B)**; MDA **(C)**; standardized regression coefficient of MDA of 37 common peaks **(D)**.

The variable influence on projection (VIP) denotes the contribution of an independent variable to a dependent variable. The larger of VIP value, the greater contribution of the independent variable to a dependent variable. VIP >1 indicates a significant contribution to the dependent variable. SOD, GSH, and MDA were used as efficacy indices for VIP (Figures 5A–C, respectively). Components with VIP >1 for SOD and GSH were intersected. MDA has a negative correlation, so coefficient analysis was carried out while projecting important variables on MDA (Figure 5D). PRR components with VIP >1 and a negative correlation were selected. Then, the PRR components with VIP >1 for SOD and GSH were intersected. Peaks 8, 11, 17, 21, and 31 had a favorable inhibitory effects on cells experiencing oxidative stress.

By cross-enriching the peaks screened by GRA and PLS-DA, peaks of 8 (paeoniflorin, galloylpaeoniflorin, albiflorin), 11 (1,2,3,4,6-o-pentagalloylglucose), 17 (benzoylpaeoniflorin), 21 (pinocembrin), and 31 (oleanic acid, isorhamnetin 3-o-neohesperidoside) were screened out, and were considered to be the preliminary Q-markers of the antioxidant bioactive of PRR.

### 3.4 Protective effects of monomeric components on cellular oxidative stress

**FIGURE 6:**
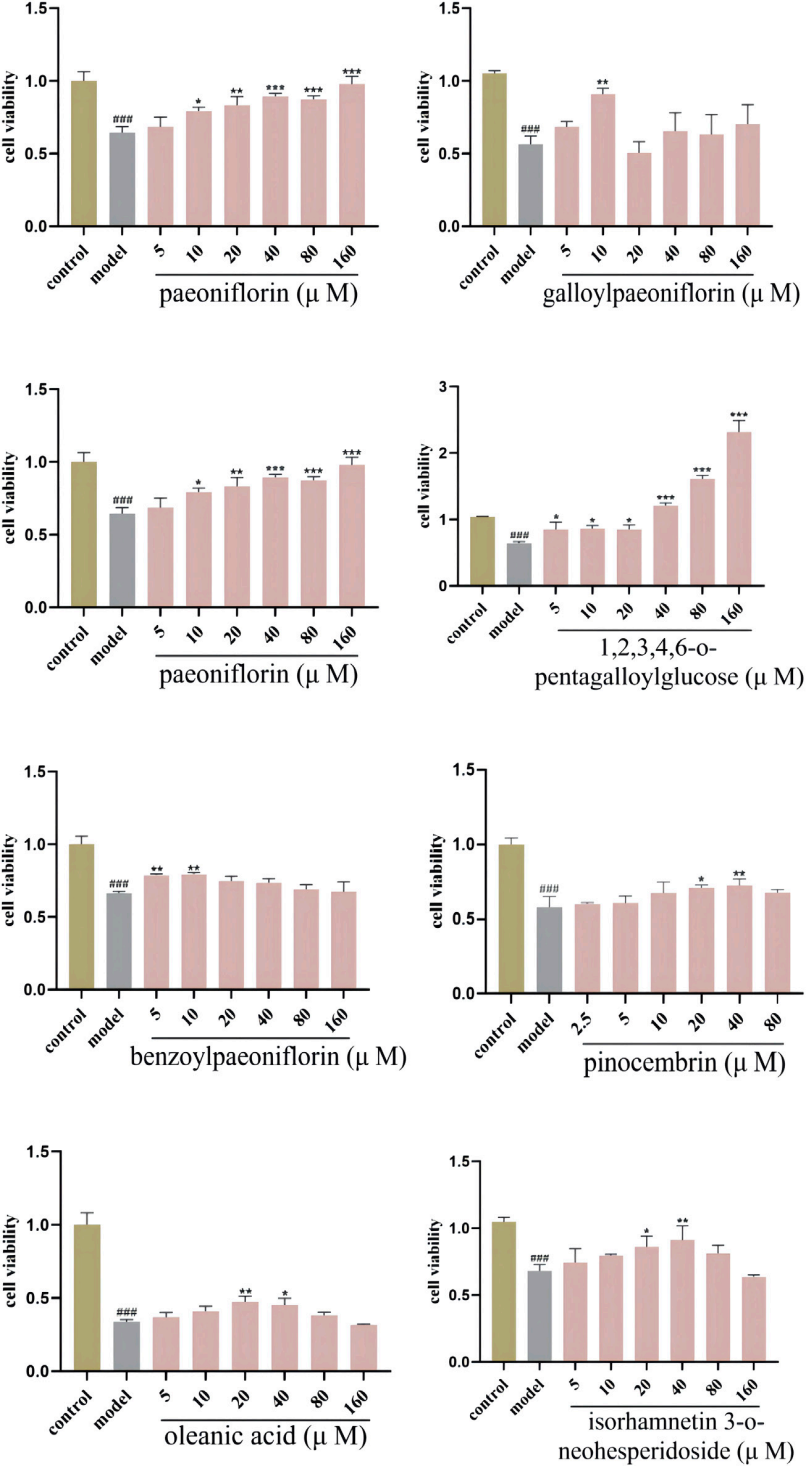
Protective effects of monomeric component on oxidant stress cell model.

To further validate the correlation between preliminary Q-markers and antioxidant bioactive, the MTT assay was applied to measure the viability of HT-22 cells. H_2_O_2_ (0.9 μM for 2 h) damaged HT-22 cells significantly, and the active components of PRR reduced the oxidative damage caused by H_2_O_2_ (Figure 6). Increasing numbers of cells survived when the concentration of paeoniflorin and 1,2,3,4,6-o-pentagalloylglucose was increased, and the minimum effective concentration was 10 μM and 5 μM, respectively. Also, benzoylpaeoniflorin (5 μM) and gallylpaeoniflorin (10 μM) had protective effects against the cellular damage under oxidative stress. Another four kinds of monomeric components also had antioxidant activities at different concentrations. These data highlighted the protective effects of PRR components on HT-22 cells injured by H_2_O_2_.

### 3.5 Potential targets screened by a PPI network

**FIGURE 7:**
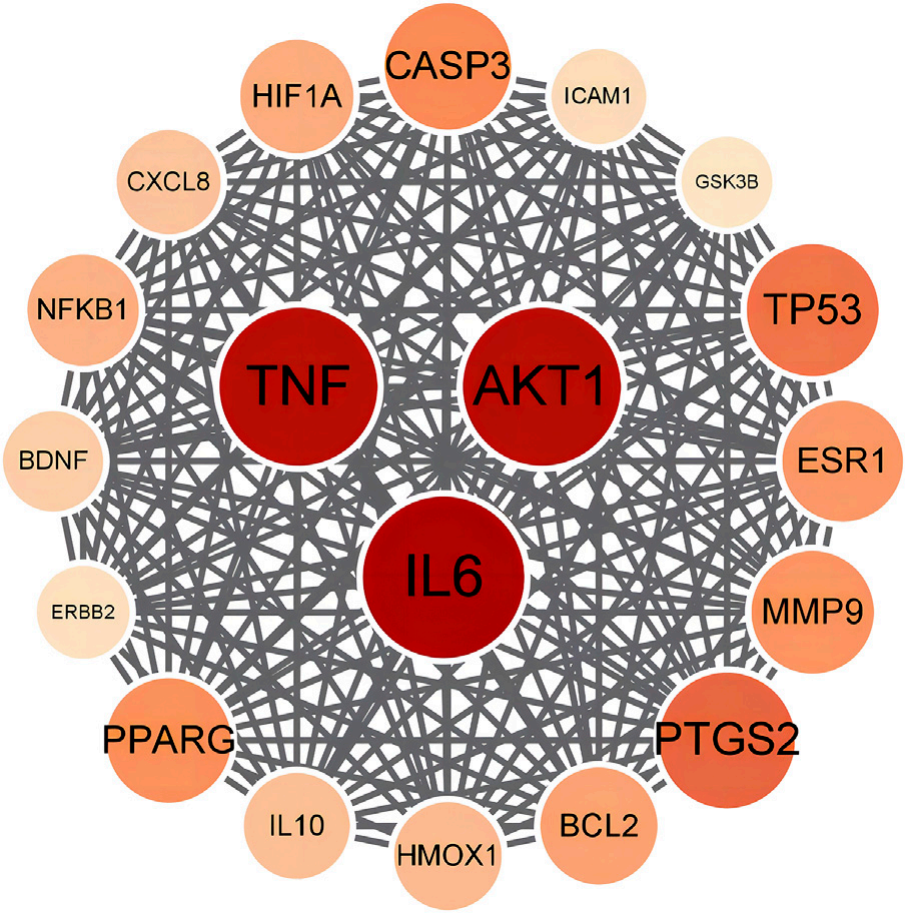
Protein-protein interaction network of PRR for antioxidant.

The chemical components of PRR were gathered from TCMSP and Pubchem databases, and the screening conditions were set as oral bioavailability ≥30% and drug likeness ≥0.18. After removing repeated and false-positive components and targets, 349 potential PRR targets were predicted. To search and integrate oxidation-related targets, databases (GeneCards, OMIM, TTD, DrugBank) were used. Repeated targets were removed, and 1740 target proteins were predicted associated with antioxidants. PRR targets were cross-referenced with oxidation targets and yielded 128 target genes. Targets with degree >50 were selected to establish a PPI network of potential targets (Figure 7). According to the degree value, the top-three targets were IL-6, AKT1, and TNF.

### 3.6 Molecular docking

**FIGURE 8:**
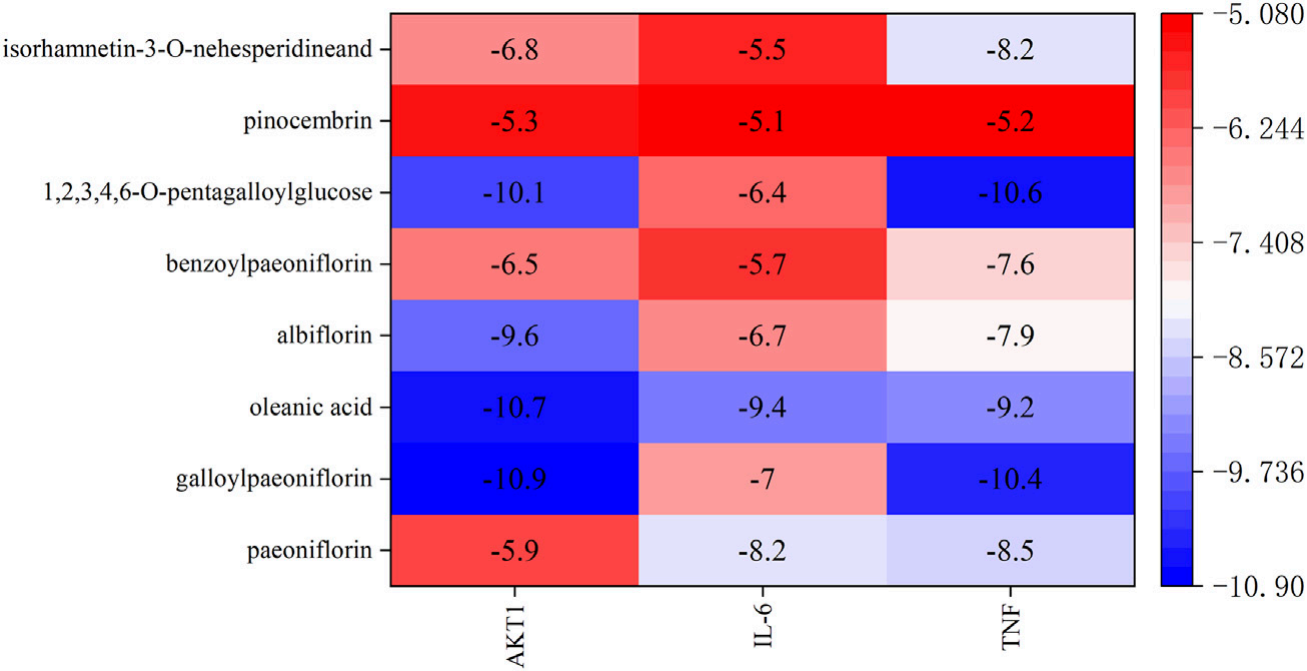
Docking energy heat map of molecular docking.

Based on the results of network pharmacology, molecular docking was undertaken to validate the binding modes of the active components and the top-three oxidation targets (AKT1, TNF, IL-6). A low binding energy indicates good binding affinity with an active site. A binding energy < −5.0 kcal/mol indicates that the drug component has “good” binding activity to a target, and a binding energy < −7 kcal/mol indicates a “strong” binding effect. Values for the binding energy are shown in Supplementary Table S4. The heat map in Figure 8 shows the binding affinity of the PRR components to the target proteins. The colors in the figure range from red to blue, and the blue range from light to dark represents the binding energy is getting smaller and smaller. The numbers in the figure are the specific values of the binding energy. It can be seen that the binding energy of the active components of PRR to the potential targets is less than −5 kcal/mol. Two-dimensional (2D) and 3D visualizations of the molecular docking of paeoniflorin, galloylpaeoniflorin, albiflorin, 1,2,3,4,6-o-pentagalloylglucose, benzoylpaeoniflorin, pinocembrin, oleanic acid, and isorhamnetin-3-o-nehesperidineand with AKT1, TNF, and IL-6 are presented in Supplementary Figure S4, the results exhibited good binding.

## 4 Discussion

The concept of Q-markers has transitioned from theory to practice, guiding a large amount of fragmented research work form an increasingly mature system. Exploring the Q-markers of TCM has become a focal point of research, but a singular method is not sufficient to ensure their scientific validity. So the integrate various methods for a comprehensive study is becoming more and more intensively. For example, the Q-markers of Astragali Radix were predicted based on network pharmacology and fingerprint (Zhang et al., 2021). Q-markers of Wenxin Formula were discoveried based on a Chinmedomics strategy (Wang Z.-W. et al., 2022). Besides, “Q-markers targeted screening” strategy were applied for comprehensive qualitative and quantitative analysis in fingerprints combined with chemometric methods (Gao et al., 2021).

As a well-known TCM, PRR is widely used in the treatment of depression (Chen et al., 2023), cerebral ischemia (Zhong et al., 2015), liver fibrosis (Song et al., 2024), and other diseases. Unfortunately, Q-markers for the antioxidant effect of PRR has not been conducted so far. The present work was carried out to find the material basis of antioxidant bioactive of PRR based on Q-Markers.

Chromatographic fingerprinting plays an important role in the quality control of TCM. It can give an overall view of the characteristics of nearly all components (Lu et al., 2022). We employed UHPLC-Q-TOF/MS to analyze the chemical components of 15 batches of PRR extracts. Chromatographic parameters (preparation of sample solution, temperature, mobile phase, detection wavelength) were optimized and thirty-seven common peaks were identified. The distribution of each common peak was, in general, stable, which supported the “specificity” and “detectability” of Q-markers. It was consistent with the “five principles” of Q-markers, also conducted to anticipate the potential Q-markers for the neuroprotective effects of JKZP (Wu Q. et al., 2024).

Oxidative stress occurs when oxidative and antioxidant components in cells are out of balance, causing cellular and tissue damage (Rotariu et al., 2022). It is gratifying that a favorable antioxidant capacity of PRR was observed by measuring the viability, activity of SOD, and levels of GSH and MDA in cells, which preliminarily confirmed the “effectiveness” of Q-markers. The effectiveness of TCM in clinical practice is directly related to the quality, which was regarded as a pivotal issue in the “five principles” of Q-marker (Jiang et al., 2018).

Grey relational analysis (GRA) is a comprehensive model of evaluation and prediction from a holistic perspective. It has a simple principle, convenient operation, and low requirements for data. By observing changes in some independent variables in different datasets, the magnitude of the association between common peaks and the corresponding efficacy data was obtained, it was also used for the spectrum-effect relationship in the research of Q-markers of antioxidant activity of Kai-Xin-San (Shan et al., 2023). However, GRA could not be used to describe the positivity or negativity of a correlation (Wu D. et al., 2024). For instance, a large negative correlation should be selected when conducting a correlation analysis for MDA. Therefore, another widely used method, partial least squares discrimination analysis (PLS-DA) was introduced. PLS-DA compensated for the deficiency of GRA, but also clarified the contribution of each component of PRR on the efficacy of PRR. Thus, combined use of GRA and PLS-DA could screen out the components that make outstanding contributions to the antioxidant effects of PRR. We attempted to use Pearson correlation analysis (Supplementary Figure S5), but no contributory components could be identified for the antioxidant activity of PRR with regard to SOD. Considering the possible differences in data processing among various methods, we incorporated and optimized the results of multimodal analysis. Eight components of PRR were found to contribute to the antioxidant effects of PRR: paeoniflorin, galloylpaeoniflorin, albiflorin, 1,2,3,4,6-o-pentagalloylglucose, benzoylpaeoniflorin, pinocembrin, oleanic acid, and isorhamnetin-3-o-nehesperidine.

Monoterpenes and their glycosides (including the four monomeric compounds of paeoniflorin, galloylpaeoniflorin, albiflorin and benzoylpaeoniflorin in PRR) possess antioxidant effects. For example, paeoniflorin has been reported to play an important part in the treatment of various diseases primarily through antioxidation (Ma et al., 2017; Lu et al., 2020; Yuan et al., 2020; Liu et al., 2021). Galloylpaeoniflorin can inhibit reactive oxygen species (ROS) production during ovariectomy-induced osteoporosis. It has a neuroprotective role by interfering with the phosphoinositide 3-kinase/AKT/nuclear factor-erythroid factor 2-related factor 2 pathway to upregulate or downregulate oxidative-stress indicators in cells and animals (Wen et al., 2018). Albiflorin has been shown to exert therapeutic effects upon enteritis by regulating levels of MDA, glutathione peroxidase, and ROS(Zhang H. et al., 2022). Benzoylpaeoniflorin has an inhibitory effect on early atherosclerosis owing to an antioxidant effect (Kim et al., 2021). The reports stated above offer further evidence that monoterpenes and their glycosides are antioxidative Q-markers.

The flavonoid pinocembrin has been shown to inhibit hepatocyte apoptosis and proinflammatory responses by reducing the ROS level (P et al., 2022). Pinocembrin has an antioxidant role and so can reduce cardiac arrhythmia and infarct size by diminishing the tissue level of MDA (Lungkaphin et al., 2015). Studies on the therapeutic effect of isorhamnetin-3-o-nehesperidine have been scarce, and studies only stay at the quantitative analysis (Xue et al., 2018). 1,2,3,4,6-o-pentagalloylglucose is a tannin. It has been reported to reduce acute lung injury mainly through anti-inflammatory and antioxidant effects. Kim et al. postulated that PRR elicits anti-atherosclerosis actions through anti-inflammatory and antioxidative effects, and that 1,2,3,4,6-o-pentagalloylglucose can reduce ROS production (Kim et al., 2021). Oleanolic acid has been used in the treatment of subarachnoid hemorrhage (Han et al., 2022), liver fibrosis (Leilei et al., 2022), diabetic nephropathy (Lee et al., 2016), and osteoarthritis (Pang et al., 2021) by inhibiting oxidative stress.

In addition to albiflorin, paeoniflorin, and 1,2,3,4,6-o-pentagalloylglucose, which we found to be major bioactive compounds in PRR, the metabolic products generated from a series of reactions in an organism must be considered. Hence, the active constituents *in vivo* may differ significantly from the inherent components of a medicinal material. For example, various metabolic products from the primitive composition of paeoniflorin, such as Paeonimetabolin I (Chen et al., 2021), oxypaeoniflorin, paeonimetabolin II glucoside (Zhang J. et al., 2022), and benzoic acid (Yu et al., 2019) have been identified in plasma. Studies have shown that Paeonimetabolin I has a significant inhibitory effect on thrombi formation via accelerating blood flow (Chen et al., 2021). Benzoic acid exerts pharmacological effects in the central nervous system by entering the bloodstream and penetrating the blood–brain barrier (Yu et al., 2019). These data suggest that some of the metabolic products from paeoniflorin have therapeutic roles in diseases, further verifying the accuracy of paeoniflorin as Q-marker.

Experimental verification is a further guarantee of the effectiveness of Q-markers. The viability of cells undergoing oxidative stress (induced by H_2_O_2_) was investigated by treatment with each monomer component at different concentrations. These monomer components displayed protective effects and indicated the reliability of screening for preliminary Q-markers.

Overall, a variety of strategies were used to study the Q-Markers of antioxidant of PRR in this paper. Although this study finally screened out chemical antioxidant Q-markers, there are also some limitations. Firstly, there is no further evidence to verify whether the antioxidant effect of eight components were equal to the whole PRR, with the possibility of some active components has been ignored. Secondly, the pharmacodynamic index of anti-oxidant was just based on the level of SOD, GSH and MDA in oxidative stress cell model, and the anti-oxidant activity *in vivo* using animal model needs to be further explored in future research. In addition, PRR also has many kinds of bio-activities include antiviral, anti-inflammatory, anti-bacterial, anti-tumor, anti-infective, except for anti-oxidant. Therefore, Q-markers that can comprehensively reflect varies kinds of biological activities of PRR need to be studied classified. Also, the negative control of Q-markers knock out from PRR experiments should be carried out, which would offer more powerful evidence for the screening results of Q-Markers.

## 5 Conclusion

Thirty-seven common peaks were identified from 15 batches of PRR by UPLC-Q-TOF-MS. Each PRR extracts could improve the survival rate of HT-22 cells suffering from H_2_O_2_-induced oxidative stress. PRR extracts could increase SOD activity and the GSH level, and reduce the MDA level, displayed a remarkable antioxidant bioactive. The preliminary antioxidant Q-markers in PRR were screened out (paeoniflorin, galloylpaeoniflorin, albiflorin, 1,2,3,4,6-o-pentagalloylglucose, benzoylpaeoniflorin, pinocembrin, oleanic acid, isorhamnetin 3-o-neohesperidoside). Experimental validation offered the evidence of protective effect of each monomer active ingredient on HT-22 cells under oxidative stress. Also, the Q-markers were all well docked with the potential antioxidant targets. Our research revealed the material basis for efficacy-oriented evaluation of PRR, as well as a reference for quality control of PRR.

## Data Availability

The original contributions presented in the study are included in the article/Supplementary Material, further inquiries can be directed to the corresponding authors.

## References

[B1] Chen C. Gong W. Tian J. Gao X. Qin X. Du G. (2023). Radix Paeoniae Alba attenuates Radix Bupleuri-induced hepatotoxicity by modulating gut microbiota to alleviate the inhibition of saikosaponins on glutathione synthetase. J. Pharm. Anal. 13, 640–659. 10.1016/j.jpha.2023.04.016 37440914 PMC10334278

[B2] Chen C. Yin Q. Tian J. Gao X. Qin X. Du G. (2021). Studies on the changes of pharmacokinetics behaviors of phytochemicals and the influence on endogenous metabolites after the combination of Radix bupleuri and Radix Paeoniae alba based on multi-component pharmacokinetics and metabolomics. Front. Pharmacol. 12, 630970. 10.3389/fphar.2021.630970 33762950 PMC7982521

[B3] Chinese Pharmacopoeia Commission (2020). Pharmacopeia of the people’s Republic of China. Beijing, China: Chinese Medical Science Press.

[B4] Feinstein W. P. Brylinski M. (2015). Calculating an optimal box size for ligand docking and virtual screening against experimental and predicted binding pockets. J. Cheminform 7, 18. 10.1186/s13321-015-0067-5 26082804 PMC4468813

[B5] Gao F.-Y. Chen H.-Y. Luo Y.-S. Chen J.-K. Yan L. Zhu J.-B. (2021). “Q-markers targeted screening” strategy for comprehensive qualitative and quantitative analysis in fingerprints of Angelica dahurica with chemometric methods. Food Chem. X 12, 100162. 10.1016/j.fochx.2021.100162 34825171 PMC8604777

[B6] Han Y. Wang C. Li X. Liang G. (2022). Oleanolic acid reduces oxidative stress and neuronal apoptosis after experimental subarachnoid hemorrhage by regulating Nrf2/HO-1 pathway. Drug Dev. Res. 83, 680–687. 10.1002/ddr.21899 34820872

[B7] He P. Zhang C. Yang Y. Tang S. Liu X. Yong J. (2023). Spectrum-effect relationships as an effective approach for quality control of natural products: a review. Molecules 28, 7011. 10.3390/molecules28207011 37894489 PMC10609026

[B8] Jiang H. Liu T. Yang Z. Ge Y. Du Y. (2020). Effects of oleanolic acid on the biological function of rat’s cardiomyocytes after hypoxia. J. China Med. Univ. 49, 230–233. 10.12007/j.issn.0258-4646.2020.03.008

[B9] Jiang Z. Yang J. Wang Y. (2018). Discrimination and identification of Q-markers based on “Spider-web” mode for quality control of traditional Chinese medicine. Phytomedicine 44, 98–102. 10.1016/j.phymed.2017.12.034 29373247

[B10] Kim C. Sim H. Bae J.-S. (2022). Benzoylpaeoniflorin activates anti-inflammatory mechanisms to mitigate sepsis in cell-culture and mouse sepsis models. Int. J. Mol. Sci. 23, 13130. 10.3390/ijms232113130 36361915 PMC9656632

[B11] Kim M. J. Kang H.-H. Seo Y. J. Kim K.-M. Kim Y.-J. Jung S. K. (2021). Paeonia lactiflora root extract and its components reduce biomarkers of early atherosclerosis via anti-inflammatory and antioxidant effects *in vitro* and *in vivo* . Antioxidants (Basel) 10, 1507. 10.3390/antiox10101507 34679642 PMC8532938

[B12] Lee E. S. Kim H. M. Kang J. S. Lee E. Y. Yadav D. Kwon M.-H. (2016). Oleanolic acid and N-acetylcysteine ameliorate diabetic nephropathy through reduction of oxidative stress and endoplasmic reticulum stress in a type 2 diabetic rat model. Nephrol. Dial. Transpl. 31, 391–400. 10.1093/ndt/gfv377 26567248

[B13] Leilei L. Wenke Q. Yuyuan L. Sihang L. Xue S. Weiqiang C. (2022). Oleanolic acid-loaded nanoparticles attenuate activation of hepatic stellate cells via suppressing TGF-β1 and oxidative stress in PM2.5-exposed hepatocytes. Toxicol. Appl. Pharmacol. 437, 115891. 10.1016/j.taap.2022.115891 35077758

[B14] Li C. Yang L. Wu H. Dai M. (2018a). Paeonol inhibits oxidized low-density lipoprotein-induced vascular endothelial cells autophagy by upregulating the expression of miRNA-30a. Front. Pharmacol. 9, 95. 10.3389/fphar.2018.00095 29472864 PMC5809422

[B15] Li S. Chu Y. Zhang R. Sun L. Chen X. (2018b). Prophylactic neuroprotection of total glucosides of Paeoniae Radix alba against semen strychni-induced neurotoxicity in rats: suppressing oxidative stress and reducing the absorption of toxic components. Nutrients 10, 514. 10.3390/nu10040514 29677121 PMC5946299

[B16] Liu C. X. Chen S. L. Xiao X. H. Zhang T. J. Liao M. L. (2016). A new concept on quality marker of Chinese materia medica: quality control for Chinese medicinal products. Chinese Traditional and Herbal Drugs. Res. Gate 47 (9), 1443–1357. 10.7501/j.issn.0253-2670.2016.09.001

[B17] Liu M.-H. Lin A.-H. Ko H.-K. Perng D.-W. Lee T.-S. Kou Y. R. (2017). Prevention of bleomycin-induced pulmonary inflammation and fibrosis in mice by paeonol. Front. Physiol. 8, 193. 10.3389/fphys.2017.00193 28408888 PMC5374202

[B18] Liu S. Li Y. Yi F. Liu Q. Chen N. He X. (2020). Resveratrol oligomers from Paeonia suffruticosa protect mice against cognitive dysfunction by regulating cholinergic, antioxidant and anti-inflammatory pathways. J. Ethnopharmacol. 260, 112983. 10.1016/j.jep.2020.112983 32442589

[B19] Liu T. Zhang N. Kong L. Chu S. Zhang T. Yan G. (2022a). Paeoniflorin alleviates liver injury in hypercholesterolemic rats through the ROCK/AMPK pathway. Front. Pharmacol. 13, 968717. 10.3389/fphar.2022.968717 36081948 PMC9445162

[B20] Liu W. Xie G. Yuan G. Xie D. Lian Z. Lin Z. (2021). 6’-O-Galloylpaeoniflorin attenuates osteoclasto-genesis and relieves ovariectomy-induced osteoporosis by inhibiting reactive oxygen species and MAPKs/c-fos/NFATc1 signaling pathway. Front. Pharmacol. 12, 641277. 10.3389/fphar.2021.641277 33897430 PMC8058459

[B21] Liu Y.-F. Zhang L. Wu Q. Feng L.-Y. (2022b). Paeoniflorin ameliorates ischemic injury in rat brain via inhibiting cytochrome c/caspase3/HDAC4 pathway. Acta Pharmacol. Sin. 43, 273–284. 10.1038/s41401-021-00671-y 33976387 PMC8791966

[B22] Lu Y.-F. Li D.-X. Zhang R. Zhao L.-L. Qiu Z. Du Y. (2022). Chemical antioxidant quality markers of Chrysanthemum morifolium using a spectrum-effect approach. Front. Pharmacol. 13, 809482. 10.3389/fphar.2022.809482 35197853 PMC8859431

[B23] Lu Y.-S. Jiang Y. Yuan J.-P. Jiang S.-B. Yang Y. Zhu P.-Y. (2020). UVA induced oxidative stress was inhibited by paeoniflorin/nrf2 signaling or PLIN2. Front. Pharmacol. 11, 736. 10.3389/fphar.2020.00736 32499710 PMC7243259

[B24] Lungkaphin A. Pongchaidecha A. Palee S. Arjinajarn P. Pompimon W. Chattipakorn N. (2015). Pinocembrin reduces cardiac arrhythmia and infarct size in rats subjected to acute myocardial ischemia/reperfusion. Appl. Physiol. Nutr. Metab. 40, 1031–1037. 10.1139/apnm-2015-0108 26319563

[B25] Ma Z. Chu L. Liu H. Wang W. Li J. Yao W. (2017). Beneficial effects of paeoniflorin on non-alcoholic fatty liver disease induced by high-fat diet in rats. Sci. Rep. 7, 44819. 10.1038/srep44819 28300221 PMC5353673

[B26] Noviana E. Indrayanto G. Rohman A. (2022). Advances in fingerprint analysis for standardization and quality control of herbal medicines. Front. Pharmacol. 13, 853023. 10.3389/fphar.2022.853023 35721184 PMC9201489

[B27] Pan C. Qian C. Chunxia S. Maohua P. Luwen W. Zuojiong G. (2022). Pinocembrin ameliorates acute liver failure via activating the Sirt1/PPARα pathway *in vitro* and *in vivo* . Eur. J. Pharmacol. 915, 174610. 10.1016/j.ejphar.2021.174610 34951978

[B28] Pang Z. Jiang Z. Zhu R. Song C. Tang H. Cao L. (2021). Bardoxolone-methyl prevents oxidative stress-mediated apoptosis and extracellular matrix degradation *in vitro* and alleviates osteoarthritis *in vivo* . Drug Des. Devel Ther. 15, 3735–3747. 10.2147/DDDT.S314767 PMC8428116 34511883

[B29] Rotariu D. Babes E. E. Tit D. M. Moisi M. Bustea C. Stoicescu M. (2022). Oxidative stress - complex pathological issues concerning the hallmark of cardiovascular and metabolic disorders. Biomed. Pharmacother. 152, 113238. 10.1016/j.biopha.2022.113238 35687909

[B30] Shan X. Yang X. Li D. Zhou L. Qin S. Li J. (2023). Research on the quality markers of antioxidant activity of Kai-Xin-San based on the spectrum-effect relationship. Front. Pharmacol. 14, 1270836. 10.3389/fphar.2023.1270836 38205371 PMC10777484

[B31] Song J. Qin B.-F. Feng Q.-Y. Zhang J.-J. Zhao G.-Y. Luo Z. (2024). Albiflorin ameliorates thioacetamide-induced hepatic fibrosis: the involvement of NURR1-mediated inflammatory signaling cascades in hepatic stellate cells activation. Ecotoxicol. Environ. Saf. 276, 116334. 10.1016/j.ecoenv.2024.116334 38626607

[B32] Tong J. Fang J. Zhu T. Xiang P. Shang J. Chen L. (2021a). Pentagalloylglucose reduces AGE-induced inflammation by activating Nrf2/HO-1 and inhibiting the JAK2/STAT3 pathway in mesangial cells. J. Pharmacol. Sci. 147, 305–314. 10.1016/j.jphs.2021.08.006 34663512

[B33] Tong N.-N. Zhou X.-Y. Peng L.-P. Liu Z.-A. Shu Q.-Y. (2021b). A comprehensive study of three species of Paeonia stem and leaf phytochemicals, and their antioxidant activities. J. Ethnopharmacol. 273, 113985. 10.1016/j.jep.2021.113985 33667571

[B34] Wang T. Tian H. Pan T. Yao S. Yu H. Wu Y. (2022a). Pinocembrin suppresses oxidized low-density lipoprotein-triggered NLRP3 inflammasome/GSDMD-mediated endothelial cell pyroptosis through an Nrf2-dependent signaling pathway. Sci. Rep. 12, 13885. 10.1038/s41598-022-18297-3 35974041 PMC9381505

[B35] Wang Z.-W. Liu C. Zhang A.-H. Yan G.-L. Sun H. Han Y. (2022b). Discovery of Q-markers of Wenxin Formula based on a Chinmedomics strategy. J. Ethnopharmacol. 298, 115576. 10.1016/j.jep.2022.115576 35963421

[B36] Wen Z. Hou W. Wu W. Zhao Y. Dong X. Bai X. (2018). 6’-O-Galloylpaeoniflorin attenuates cerebral ischemia reperfusion-induced neuroinflammation and oxidative stress via PI3K/Akt/Nrf2 activation. Oxid. Med. Cell Longev. 2018, 8678267. 10.1155/2018/8678267 29765506 PMC5889897

[B37] Wu D. Lin X. Liu K. Ning H. Luo W. Zhao G. (2024a). Recognition of antitussive components in Farfarae Flos based on grey relational analysis and partial least squares regression. Zhong Nan Da Xue Xue Bao Yi Xue Ban. 49, 435–446. 10.11817/j.issn.1672-7347.2024.230344 38970518 PMC11208408

[B38] Wu Q. Ou C. Wang J. Wu X. Gao Z. Zhao Y. (2024b). Jiawei Kongsheng Zhenzhong Pill: marker compounds, absorption into the serum (rat), and Q-markers identified by UPLC-Q-TOF-MS/MS. Front. Pharmacol. 15, 1328632. 10.3389/fphar.2024.1328632 38375037 PMC10875140

[B39] Xu J.-J. Xu F. Wang W. Zhang Y.-F. Hao B.-Q. Shang M.-Y. (2022). Elucidation of the mechanisms and effective substances of Paeoniae Radix Rubra against toxic heat and blood stasis syndrome with a stage-oriented strategy. Front. Pharmacol. 13, 842839. 10.3389/fphar.2022.842839 35308239 PMC8931751

[B40] Xue H. Jiang J. Zhang Y. Meng X.-T. Xue A. Qiao Y. (2023). Mechanism of albiflorin in improvement of Alzheimer’s disease based on network pharmacology and *in vitro* experiments. Zhongguo Zhong Yao Za Zhi 48, 4738–4746. 10.19540/j.cnki.cjcmm.20230510.701 37802813

[B41] Xue J. Zhi-cheng W. Bin D. Xian-gang L. Chang-yu Y. Yue-ran Z. (2018). Simultaneous determination of typhaneoside and isorhamnetin-3-O-neohesperidoside in Tianqi Tongjing capsules by UPLC-MS/MS. Chin. J. Pharm. Analysis 38, 1055–1060. 10.16155/j.0254-1793.2018.06.20

[B42] Ye S. Mao B. Yang L. Fu W. Hou J. (2016). Thrombosis recanalization by paeoniflorin through the upregulation of urokinase-type plasminogen activator via the MAPK signaling pathway. Mol. Med. Rep. 13, 4593–4598. 10.3892/mmr.2016.5146 27082639 PMC4878539

[B43] Yu J.-B. Zhao Z.-X. Peng R. Pan L.-B. Fu J. Ma S.-R. (2019). Gut microbiota-based pharmacokinetics and the antidepressant mechanism of paeoniflorin. Front. Pharmacol. 10, 268. 10.3389/fphar.2019.00268 30949054 PMC6435784

[B44] Yuan J. Lu Y. Wang H. Feng Y. Jiang S. Gao X.-H. (2020). Paeoniflorin resists H2O2-induced oxidative stress in melanocytes by JNK/Nrf2/HO-1 pathway. Front. Pharmacol. 11, 536. 10.3389/fphar.2020.00536 32410998 PMC7198857

[B45] Zhang H. Wang J. Lang W. Liu H. Zhang Z. Wu T. (2022a). Albiflorin ameliorates inflammation and oxidative stress by regulating the NF-κB/NLRP3 pathway in Methotrexate-induced enteritis. Int. Immunopharmacol. 109, 108824. 10.1016/j.intimp.2022.108824 35561481

[B46] Zhang J. Lv Y. Zhang J. Shi W.-J. Guo X.-Y. Xu J.-J. (2022b). Metabolism of Paeoniae Radix Rubra and its 14 constituents in mice. Front. Pharmacol. 13, 995641. 10.3389/fphar.2022.995641 36267278 PMC9577399

[B47] Zhang L. Wei W. (2020). Anti-inflammatory and immunoregulatory effects of paeoniflorin and total glucosides of paeony. Pharmacol. Ther. 207, 107452. 10.1016/j.pharmthera.2019.107452 31836457

[B48] Zhang S.-J. Zhang Y.-G. Li D.-H. Wu H.-W. Niu J.-T. Si X.-L. (2021). Prediction of Q-markers of Astragali Radix based on network pharmacology and fingerprint. Zhongguo Zhong Yao Za Zhi 46, 2691–2698. 10.19540/j.cnki.cjcmm.20200925.201 34296565

[B49] Zhao F. Peng C. Li H. Chen H. Yang Y. Ai Q. (2023). Paeoniae Radix Rubra extract attenuates cerebral ischemia injury by inhibiting ferroptosis and activating autophagy through the PI3K/Akt signalling pathway. J. Ethnopharmacol. 315, 116567. 10.1016/j.jep.2023.116567 37172921

[B50] Zhao Y. Wan H. Yang J. Huang Y. He Y. Wan H. (2022). Ultrasound-assisted preparation of “Ready-to-use” extracts from Radix Paeoniae Rubra with natural deep eutectic solvents and neuroprotectivity evaluation of the extracts against cerebral ischemic/reperfusion injury. Ultrason. Sonochem 84, 105968. 10.1016/j.ultsonch.2022.105968 35272238 PMC8908277

[B51] Zhong M. Song W. Xu Y. Ye Y. Feng L. (2015). Paeoniflorin ameliorates ischemic neuronal damage *in vitro* via adenosine A1 receptor-mediated transactivation of epidermal growth factor receptor. Acta Pharmacol. Sin. 36, 298–310. 10.1038/aps.2014.154 25661317 PMC4349929

