# Supplementary material for: Screening of potential antioxidant bioactive Q-markers of paeoniae radix rubra based on an integrated multimodal strategy
Source: Front Pharmacol. 2024 Aug 15;15:1447959. doi: 10.3389/fphar.2024.1447959 (PMC11357914; doi:10.3389/fphar.2024.1447959)
Supplement: Supplementary file 1 [file DataSheet1.docx]

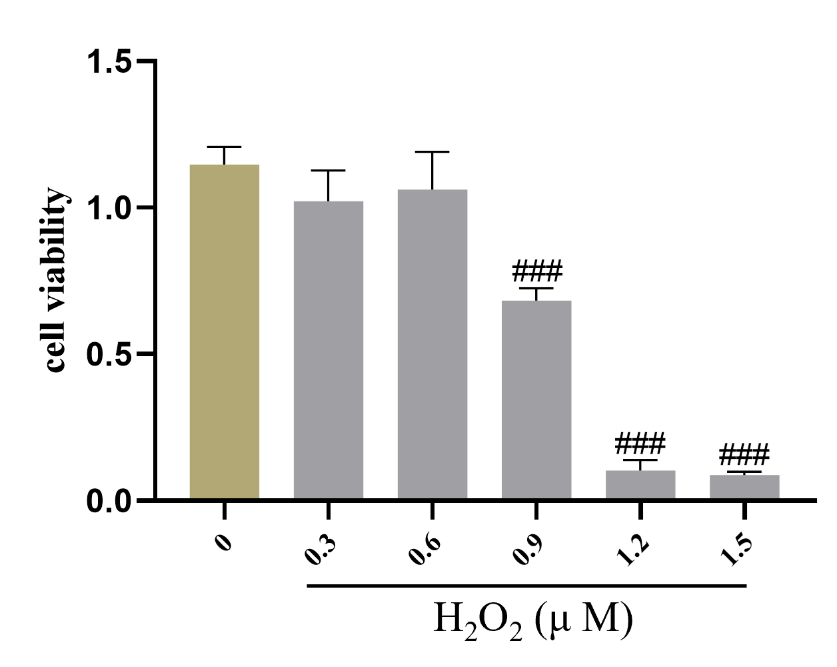


Figure S1. Effect of different concentrations of H_2_O_2_ on cell survival rate

|  |  |
| --- | --- |
| A | B |

Figure S2. UPLC-QTOF-MS total ion chromatography in positive ion (A) and negative ion (B) mode





Figure S3. Heat map of principal components and differential components of 15 Batches of PRR.

Table S1. Grey correlation coefficient between 37 common peaks of PRR and SOD

| peak number | Sample number | | | | | | | | | | | | | | | |
| --- | --- | --- | --- | --- | --- | --- | --- | --- | --- | --- | --- | --- | --- | --- | --- | --- |
|  | S1 | S2 | S3 | S4 | S5 | S6 | S7 | S8 | S9 | S10 | S11 | S12 | S13 | S14 | S15 | average |
| X1 | 0.92 | 0.92 | 0.95 | 0.96 | 0.93 | 0.97 | 0.95 | 0.88 | 0.89 | 1.00 | 1.00 | 0.97 | 1.00 | 0.92 | 0.96 | 0.95 |
| X2 | 0.83 | 0.87 | 0.86 | 0.78 | 0.94 | 0.97 | 1.00 | 0.91 | 0.87 | 0.99 | 0.94 | 0.95 | 0.95 | 0.94 | 0.98 | 0.92 |
| X3 | 0.95 | 0.99 | 0.84 | 0.78 | 0.88 | 0.95 | 0.94 | 0.81 | 0.96 | 0.84 | 0.90 | 0.99 | 0.89 | 0.89 | 0.97 | 0.91 |
| X4 | 0.55 | 0.88 | 1.00 | 0.92 | 0.91 | 0.92 | 0.92 | 0.81 | 0.92 | 0.82 | 0.69 | 0.80 | 0.81 | 0.82 | 0.87 | 0.84 |
| X5 | 0.89 | 0.95 | 0.86 | 0.96 | 0.96 | 0.99 | 0.95 | 1.00 | 1.00 | 0.87 | 0.82 | 1.00 | 0.84 | 0.99 | 0.80 | 0.93 |
| X6 | 0.72 | 0.99 | 0.94 | 0.68 | 0.86 | 0.85 | 0.97 | 0.92 | 0.81 | 0.93 | 0.90 | 0.77 | 0.80 | 0.85 | 0.86 | 0.86 |
| X7 | 0.76 | 0.91 | 0.93 | 0.55 | 0.81 | 0.83 | 0.89 | 0.77 | 0.91 | 0.86 | 0.60 | 0.77 | 0.86 | 0.83 | 0.84 | 0.81 |
| X8 | 0.93 | 0.99 | 0.95 | 0.91 | 0.81 | 0.91 | 0.95 | 0.92 | 0.84 | 0.91 | 0.84 | 0.97 | 0.93 | 0.73 | 1.00 | 0.91 |
| X9 | 0.89 | 0.75 | 0.81 | 0.84 | 0.98 | 0.95 | 0.95 | 0.86 | 0.83 | 0.86 | 0.85 | 0.96 | 0.96 | 0.83 | 0.75 | 0.87 |
| X10 | 0.76 | 0.87 | 0.93 | 0.88 | 0.91 | 0.96 | 0.99 | 0.97 | 0.96 | 0.98 | 0.98 | 0.87 | 0.96 | 0.85 | 0.98 | 0.92 |
| X11 | 0.92 | 0.91 | 0.96 | 0.95 | 0.93 | 0.88 | 0.99 | 0.95 | 0.97 | 0.98 | 0.95 | 0.89 | 0.98 | 1.00 | 0.98 | 0.95 |
| X12 | 0.95 | 0.88 | 1.00 | 0.83 | 0.99 | 0.85 | 0.85 | 0.96 | 0.86 | 0.90 | 0.83 | 0.88 | 0.91 | 0.76 | 0.81 | 0.88 |
| X13 | 0.61 | 0.96 | 0.95 | 0.92 | 0.88 | 0.87 | 0.99 | 0.99 | 0.85 | 0.91 | 0.89 | 0.83 | 0.94 | 0.88 | 0.79 | 0.88 |
| X14 | 0.58 | 0.91 | 0.67 | 0.46 | 0.79 | 0.79 | 0.81 | 0.75 | 0.93 | 0.76 | 0.85 | 0.74 | 0.77 | 0.79 | 0.81 | 0.76 |
| X15 | 0.47 | 0.93 | 0.93 | 0.76 | 0.81 | 0.84 | 0.85 | 0.80 | 0.98 | 0.77 | 0.56 | 0.75 | 0.80 | 0.86 | 0.84 | 0.80 |
| X16 | 0.55 | 0.99 | 0.76 | 0.96 | 0.81 | 0.89 | 0.85 | 0.99 | 0.66 | 0.80 | 0.83 | 0.82 | 0.84 | 0.86 | 0.87 | 0.83 |
| X17 | 0.98 | 0.90 | 0.91 | 0.87 | 0.96 | 0.95 | 0.89 | 0.95 | 0.96 | 0.99 | 0.98 | 0.85 | 0.93 | 1.00 | 0.89 | 0.93 |
| X18 | 0.33 | 0.91 | 0.79 | 0.79 | 0.81 | 0.79 | 0.82 | 0.76 | 0.74 | 0.79 | 0.84 | 0.75 | 0.78 | 0.80 | 0.81 | 0.77 |
| X19 | 0.60 | 0.99 | 0.84 | 0.78 | 0.87 | 0.87 | 0.90 | 0.80 | 0.70 | 0.88 | 0.78 | 0.83 | 0.89 | 0.85 | 0.88 | 0.83 |
| X20 | 0.74 | 1.00 | 0.84 | 0.74 | 0.88 | 0.93 | 0.93 | 0.76 | 0.97 | 0.95 | 0.88 | 0.91 | 0.91 | 0.92 | 0.97 | 0.89 |
| X21 | 0.93 | 0.91 | 0.83 | 0.99 | 0.89 | 0.99 | 0.99 | 0.91 | 0.87 | 0.99 | 0.94 | 0.93 | 0.97 | 0.90 | 0.99 | 0.93 |
| X22 | 0.91 | 1.00 | 0.90 | 0.66 | 0.82 | 0.82 | 0.93 | 0.82 | 0.67 | 0.95 | 0.72 | 0.85 | 0.93 | 0.83 | 0.87 | 0.84 |
| X23 | 0.94 | 0.90 | 0.88 | 0.95 | 0.92 | 0.93 | 0.88 | 0.93 | 0.83 | 0.83 | 0.87 | 0.93 | 1.00 | 0.96 | 1.00 | 0.92 |
| X24 | 0.94 | 0.88 | 0.99 | 0.92 | 0.95 | 0.97 | 0.97 | 0.86 | 0.94 | 0.93 | 0.87 | 0.88 | 1.00 | 0.94 | 0.98 | 0.94 |
| X25 | 1.00 | 0.92 | 0.78 | 0.55 | 0.94 | 0.88 | 0.91 | 0.79 | 0.89 | 1.00 | 0.56 | 0.81 | 0.95 | 0.85 | 0.83 | 0.84 |
| X26 | 0.98 | 0.82 | 0.91 | 0.81 | 0.90 | 1.00 | 0.96 | 0.99 | 0.92 | 0.96 | 0.92 | 0.91 | 0.95 | 0.92 | 0.97 | 0.93 |
| X27 | 0.95 | 0.99 | 0.91 | 0.58 | 0.88 | 0.96 | 0.91 | 0.84 | 0.91 | 0.93 | 0.76 | 0.84 | 0.92 | 0.92 | 0.88 | 0.88 |
| X28 | 0.98 | 0.96 | 0.95 | 0.86 | 0.90 | 0.90 | 0.99 | 0.85 | 0.78 | 1.00 | 0.91 | 0.89 | 0.96 | 0.97 | 0.99 | 0.93 |
| X29 | 0.92 | 0.94 | 0.96 | 0.93 | 0.85 | 0.87 | 0.95 | 0.85 | 0.58 | 0.91 | 0.97 | 0.94 | 0.91 | 0.97 | 0.98 | 0.90 |
| X30 | 0.84 | 0.99 | 0.84 | 0.72 | 0.95 | 0.91 | 0.99 | 0.86 | 0.94 | 1.00 | 0.97 | 0.90 | 0.93 | 0.90 | 0.96 | 0.91 |
| X31 | 0.97 | 0.97 | 0.93 | 0.86 | 0.87 | 0.91 | 0.97 | 0.86 | 0.79 | 0.96 | 0.87 | 0.87 | 0.99 | 0.96 | 0.94 | 0.91 |
| X32 | 0.88 | 0.93 | 0.98 | 0.92 | 0.81 | 0.90 | 0.96 | 0.83 | 0.69 | 0.94 | 0.85 | 0.87 | 0.87 | 0.99 | 0.95 | 0.89 |
| X33 | 0.97 | 0.88 | 0.71 | 0.85 | 0.80 | 0.93 | 0.82 | 0.99 | 0.95 | 0.86 | 0.86 | 1.00 | 0.93 | 0.93 | 0.85 | 0.89 |
| X34 | 0.93 | 0.89 | 0.91 | 0.98 | 0.99 | 0.94 | 0.93 | 0.99 | 0.99 | 0.88 | 0.97 | 0.87 | 0.98 | 0.99 | 0.92 | 0.94 |
| X35 | 0.92 | 0.97 | 0.73 | 0.98 | 0.90 | 0.88 | 0.99 | 0.81 | 0.61 | 0.97 | 0.90 | 0.87 | 0.97 | 0.84 | 0.88 | 0.88 |
| X36 | 0.94 | 0.74 | 0.92 | 0.81 | 0.94 | 0.87 | 0.90 | 0.94 | 0.99 | 0.94 | 0.99 | 0.91 | 0.99 | 0.93 | 0.93 | 0.92 |
| X37 | 0.97 | 0.89 | 0.73 | 0.86 | 0.80 | 0.95 | 0.89 | 0.98 | 0.94 | 0.87 | 0.82 | 0.91 | 0.93 | 0.98 | 0.81 | 0.89 |

Table S2. Grey correlation coefficient between 37 common peaks of PRR and GSH

| peak number | Sample number | | | | | | | | | | | | | | | | |
| --- | --- | --- | --- | --- | --- | --- | --- | --- | --- | --- | --- | --- | --- | --- | --- | --- | --- |
|  | S1 | S2 | S3 | S4 | S5 | S6 | S7 | S8 | S9 | S10 | S11 | S12 | S13 | S14 | S15 | average | |
| X1 | 0.97 | 0.92 | 0.85 | 0.92 | 0.99 | 0.99 | 0.90 | 0.99 | 0.76 | 0.96 | 0.94 | 0.95 | 0.96 | 0.99 | 0.89 | 0.93 |  |
| X2 | 0.91 | 0.86 | 0.77 | 0.75 | 0.99 | 0.94 | 0.95 | 0.97 | 0.74 | 0.95 | 0.99 | 0.94 | 0.98 | 0.97 | 0.95 | 0.91 |  |
| X3 | 0.94 | 0.99 | 0.76 | 0.75 | 0.93 | 0.92 | 0.99 | 0.90 | 0.81 | 0.80 | 0.85 | 0.98 | 0.92 | 0.97 | 0.90 | 0.89 |  |
| X4 | 0.57 | 0.87 | 0.89 | 0.89 | 0.85 | 0.89 | 0.96 | 0.90 | 0.91 | 0.84 | 0.65 | 0.79 | 0.83 | 0.88 | 0.93 | 0.84 |  |
| X5 | 0.80 | 0.95 | 0.78 | 0.99 | 0.90 | 0.96 | 1.00 | 0.88 | 0.84 | 0.83 | 0.85 | 0.99 | 0.80 | 0.90 | 0.74 | 0.88 |  |
| X6 | 0.78 | 0.98 | 0.95 | 0.66 | 0.80 | 0.82 | 0.92 | 0.96 | 0.94 | 0.89 | 0.85 | 0.75 | 0.81 | 0.92 | 0.91 | 0.86 |  |
| X7 | 0.82 | 0.91 | 0.96 | 0.53 | 0.76 | 0.80 | 0.92 | 0.85 | 0.93 | 0.88 | 0.57 | 0.75 | 0.88 | 0.90 | 0.90 | 0.82 |  |
| X8 | 0.96 | 0.99 | 0.94 | 0.93 | 0.76 | 0.89 | 0.90 | 0.82 | 0.99 | 0.95 | 0.88 | 0.95 | 0.96 | 0.67 | 0.93 | 0.90 |  |
| X9 | 0.80 | 0.74 | 0.73 | 0.86 | 0.91 | 0.97 | 0.90 | 0.77 | 0.72 | 0.88 | 0.88 | 0.94 | 0.93 | 0.76 | 0.70 | 0.83 |  |
| X10 | 0.83 | 0.87 | 0.83 | 0.85 | 0.85 | 0.92 | 0.94 | 0.91 | 0.88 | 0.98 | 0.93 | 0.86 | 1.00 | 0.92 | 0.94 | 0.90 |  |
| X11 | 0.97 | 0.90 | 0.86 | 0.91 | 0.86 | 0.90 | 0.96 | 0.93 | 0.87 | 0.98 | 1.00 | 0.87 | 0.98 | 0.91 | 0.94 | 0.92 |  |
| X12 | 0.85 | 0.88 | 0.89 | 0.85 | 0.93 | 0.87 | 0.80 | 0.92 | 0.74 | 0.93 | 0.86 | 0.86 | 0.94 | 0.70 | 0.75 | 0.85 |  |
| X13 | 0.65 | 0.96 | 0.94 | 0.94 | 0.82 | 0.84 | 0.96 | 0.88 | 0.73 | 0.94 | 0.94 | 0.81 | 0.97 | 0.80 | 0.73 | 0.86 |  |
| X14 | 0.61 | 0.90 | 0.71 | 0.44 | 0.74 | 0.76 | 0.83 | 0.82 | 0.90 | 0.78 | 0.80 | 0.72 | 0.79 | 0.85 | 0.86 | 0.77 |  |
| X15 | 0.49 | 0.92 | 0.83 | 0.73 | 0.76 | 0.81 | 0.88 | 0.88 | 0.86 | 0.78 | 0.52 | 0.73 | 0.82 | 0.94 | 0.90 | 0.79 |  |
| X16 | 0.57 | 0.99 | 0.82 | 0.92 | 0.75 | 0.86 | 0.88 | 0.88 | 0.74 | 0.81 | 0.86 | 0.80 | 0.87 | 0.93 | 0.93 | 0.84 |  |
| X17 | 0.91 | 0.90 | 0.81 | 0.84 | 0.98 | 0.97 | 0.85 | 0.93 | 0.88 | 0.95 | 0.97 | 0.84 | 0.96 | 0.91 | 0.95 | 0.91 |  |
| X18 | 0.33 | 0.90 | 0.72 | 0.76 | 0.76 | 0.76 | 0.84 | 0.83 | 0.85 | 0.81 | 0.80 | 0.73 | 0.79 | 0.86 | 0.86 | 0.77 |  |
| X19 | 0.63 | 0.99 | 0.75 | 0.75 | 0.81 | 0.84 | 0.93 | 0.89 | 0.79 | 0.91 | 0.74 | 0.81 | 0.92 | 0.92 | 0.94 | 0.84 |  |
| X20 | 0.80 | 1.00 | 0.75 | 0.71 | 0.82 | 0.90 | 0.89 | 0.84 | 0.82 | 0.99 | 0.83 | 0.90 | 0.87 | 1.00 | 0.95 | 0.87 |  |
| X21 | 0.96 | 0.90 | 0.75 | 0.95 | 0.84 | 0.96 | 0.94 | 0.98 | 0.98 | 0.97 | 0.88 | 0.91 | 1.00 | 0.82 | 0.92 | 0.92 |  |
| X22 | 0.99 | 1.00 | 0.80 | 0.63 | 0.77 | 0.79 | 0.98 | 0.91 | 0.75 | 0.98 | 0.68 | 0.83 | 0.96 | 0.90 | 0.93 | 0.86 |  |
| X23 | 0.95 | 0.90 | 0.79 | 0.98 | 0.86 | 0.90 | 0.83 | 0.83 | 0.71 | 0.79 | 0.90 | 0.93 | 0.96 | 0.88 | 0.92 | 0.88 |  |
| X24 | 0.95 | 0.87 | 0.90 | 0.89 | 0.89 | 0.94 | 0.92 | 0.96 | 0.89 | 0.97 | 0.82 | 0.87 | 0.96 | 0.97 | 0.95 | 0.92 |  |
| X25 | 0.89 | 0.92 | 0.70 | 0.53 | 0.87 | 0.85 | 0.95 | 0.87 | 0.76 | 0.95 | 0.52 | 0.79 | 0.91 | 0.92 | 0.88 | 0.82 |  |
| X26 | 0.87 | 0.81 | 0.81 | 0.78 | 0.84 | 0.97 | 0.91 | 0.90 | 0.78 | 0.92 | 0.97 | 0.89 | 0.98 | 0.84 | 0.96 | 0.88 |  |
| X27 | 0.85 | 0.99 | 0.82 | 0.55 | 0.93 | 0.93 | 0.95 | 0.94 | 0.77 | 0.97 | 0.72 | 0.82 | 0.95 | 1.00 | 0.94 | 0.87 |  |
| X28 | 0.87 | 0.96 | 0.85 | 0.83 | 0.84 | 0.87 | 0.94 | 0.94 | 0.90 | 0.96 | 0.86 | 0.87 | 0.92 | 0.93 | 0.94 | 0.90 |  |
| X29 | 0.83 | 0.94 | 0.86 | 0.95 | 0.79 | 0.84 | 0.99 | 0.95 | 0.64 | 0.87 | 0.92 | 0.92 | 0.87 | 0.94 | 0.94 | 0.88 |  |
| X30 | 0.92 | 0.99 | 0.76 | 0.69 | 0.89 | 0.88 | 0.96 | 0.96 | 0.90 | 0.96 | 0.92 | 0.89 | 0.89 | 0.98 | 0.97 | 0.90 |  |
| X31 | 0.92 | 0.96 | 0.96 | 0.82 | 0.81 | 0.88 | 0.98 | 0.96 | 0.91 | 0.92 | 0.82 | 0.85 | 0.97 | 0.94 | 0.98 | 0.91 |  |
| X32 | 0.79 | 0.93 | 0.88 | 0.88 | 0.76 | 0.88 | 0.91 | 0.92 | 0.78 | 0.90 | 0.80 | 0.85 | 0.83 | 0.92 | 0.98 | 0.87 |  |
| X33 | 0.86 | 0.87 | 0.77 | 0.87 | 0.75 | 0.90 | 0.78 | 0.90 | 0.81 | 0.89 | 0.90 | 0.99 | 0.96 | 0.85 | 0.79 | 0.86 |  |
| X34 | 0.84 | 0.88 | 0.82 | 0.95 | 0.93 | 0.97 | 0.98 | 0.90 | 0.84 | 0.85 | 0.98 | 0.85 | 0.94 | 0.90 | 0.85 | 0.90 |  |
| X35 | 0.82 | 0.97 | 0.79 | 0.98 | 0.84 | 0.85 | 0.95 | 0.90 | 0.68 | 0.99 | 0.85 | 0.85 | 0.94 | 0.90 | 0.94 | 0.88 |  |
| X36 | 0.84 | 0.73 | 0.82 | 0.83 | 0.88 | 0.89 | 0.94 | 0.83 | 0.85 | 0.98 | 0.96 | 0.89 | 0.95 | 0.84 | 0.86 | 0.87 |  |
| X37 | 0.87 | 0.89 | 0.79 | 0.87 | 0.75 | 0.98 | 0.84 | 0.90 | 0.80 | 0.90 | 0.85 | 0.92 | 0.96 | 0.93 | 0.75 | 0.87 |  |

Table S3. Grey correlation coefficient between 37 common peaks of PRR and MDA

| peak number | Sample number | | | | | | | | | | | | | | | |
| --- | --- | --- | --- | --- | --- | --- | --- | --- | --- | --- | --- | --- | --- | --- | --- | --- |
|  | S1 | S2 | S3 | S4 | S5 | S6 | S7 | S8 | S9 | S10 | S11 | S12 | S13 | S14 | S15 | average |
| X1 | 0.93 | 0.80 | 0.96 | 0.93 | 0.95 | 0.91 | 0.94 | 0.90 | 0.98 | 0.97 | 0.83 | 0.93 | 0.94 | 0.98 | 0.88 | 0.92 |
| X2 | 0.84 | 0.84 | 0.94 | 0.76 | 0.96 | 0.97 | 1.00 | 0.94 | 0.99 | 0.98 | 0.88 | 0.92 | 0.89 | 0.96 | 0.83 | 0.91 |
| X3 | 0.97 | 0.75 | 0.91 | 0.76 | 0.89 | 0.98 | 0.95 | 0.83 | 0.91 | 0.86 | 0.76 | 0.96 | 0.84 | 0.98 | 0.87 | 0.88 |
| X4 | 0.55 | 0.83 | 0.91 | 0.90 | 0.89 | 0.98 | 0.92 | 0.83 | 0.81 | 0.80 | 0.60 | 0.78 | 0.77 | 0.89 | 0.75 | 0.82 |
| X5 | 0.88 | 0.78 | 0.94 | 0.99 | 0.95 | 0.95 | 0.96 | 0.97 | 0.87 | 0.89 | 0.98 | 0.97 | 0.89 | 0.90 | 0.93 | 0.92 |
| X6 | 0.73 | 0.74 | 0.86 | 0.67 | 0.84 | 0.90 | 0.97 | 0.94 | 0.72 | 0.96 | 0.76 | 0.74 | 0.75 | 0.93 | 0.74 | 0.82 |
| X7 | 0.76 | 0.70 | 0.86 | 0.54 | 0.80 | 0.88 | 0.89 | 0.79 | 0.80 | 0.84 | 0.53 | 0.75 | 0.81 | 0.91 | 0.73 | 0.77 |
| X8 | 0.95 | 0.75 | 0.87 | 0.93 | 0.80 | 0.97 | 0.94 | 0.90 | 0.75 | 0.89 | 0.99 | 0.94 | 0.87 | 0.68 | 0.85 | 0.87 |
| X9 | 0.88 | 0.99 | 0.88 | 0.86 | 0.96 | 0.89 | 0.95 | 0.84 | 0.95 | 0.84 | 0.98 | 0.92 | 0.97 | 0.77 | 0.87 | 0.90 |
| X10 | 0.77 | 0.84 | 0.98 | 0.86 | 0.89 | 0.98 | 0.98 | 1.00 | 0.84 | 0.95 | 0.82 | 0.85 | 0.90 | 0.93 | 0.83 | 0.90 |
| X11 | 0.93 | 0.81 | 0.95 | 0.92 | 0.91 | 0.83 | 1.00 | 0.98 | 0.85 | 0.95 | 0.87 | 0.86 | 0.92 | 0.90 | 0.84 | 0.90 |
| X12 | 0.94 | 0.83 | 0.91 | 0.85 | 0.98 | 0.80 | 0.84 | 0.99 | 0.98 | 0.88 | 0.99 | 0.85 | 0.85 | 0.71 | 0.95 | 0.89 |
| X13 | 0.62 | 0.73 | 0.87 | 0.94 | 0.86 | 0.92 | 1.00 | 0.96 | 0.97 | 0.88 | 0.92 | 0.80 | 0.88 | 0.80 | 0.91 | 0.87 |
| X14 | 0.59 | 0.69 | 0.62 | 0.45 | 0.78 | 0.83 | 0.81 | 0.77 | 0.82 | 0.75 | 0.73 | 0.72 | 0.73 | 0.86 | 0.71 | 0.72 |
| X15 | 0.48 | 0.71 | 0.98 | 0.75 | 0.80 | 0.88 | 0.85 | 0.82 | 0.85 | 0.75 | 0.50 | 0.73 | 0.76 | 0.95 | 0.73 | 0.77 |
| X16 | 0.55 | 0.75 | 0.71 | 0.93 | 0.79 | 0.95 | 0.85 | 0.96 | 0.60 | 0.78 | 1.00 | 0.80 | 0.80 | 0.95 | 0.75 | 0.81 |
| X17 | 1.00 | 0.81 | 0.99 | 0.85 | 0.97 | 0.89 | 0.89 | 0.98 | 0.84 | 0.98 | 0.85 | 0.83 | 0.87 | 0.91 | 0.77 | 0.90 |
| X18 | 0.33 | 0.69 | 0.86 | 0.77 | 0.80 | 0.83 | 0.82 | 0.78 | 0.67 | 0.77 | 0.72 | 0.72 | 0.74 | 0.87 | 0.71 | 0.74 |
| X19 | 0.60 | 0.74 | 0.91 | 0.77 | 0.85 | 0.92 | 0.90 | 0.82 | 0.64 | 0.86 | 0.68 | 0.80 | 0.84 | 0.94 | 0.76 | 0.80 |
| X20 | 0.75 | 0.75 | 0.91 | 0.73 | 0.87 | 0.99 | 0.93 | 0.78 | 0.89 | 0.92 | 0.75 | 0.89 | 0.97 | 0.99 | 0.83 | 0.86 |
| X21 | 0.94 | 0.81 | 0.91 | 0.96 | 0.88 | 0.94 | 0.99 | 0.93 | 0.77 | 0.96 | 0.79 | 0.90 | 0.91 | 0.82 | 0.86 | 0.89 |
| X22 | 0.92 | 0.75 | 0.98 | 0.64 | 0.81 | 0.87 | 0.94 | 0.84 | 0.61 | 0.92 | 0.63 | 0.82 | 0.87 | 0.91 | 0.75 | 0.82 |
| X23 | 0.96 | 0.82 | 0.97 | 0.98 | 0.91 | 0.99 | 0.87 | 0.91 | 0.95 | 0.85 | 0.96 | 0.96 | 0.94 | 0.88 | 0.85 | 0.92 |
| X24 | 0.96 | 0.84 | 0.91 | 0.90 | 0.94 | 0.96 | 0.97 | 0.88 | 0.83 | 0.91 | 0.74 | 0.86 | 0.94 | 0.96 | 0.83 | 0.89 |
| X25 | 0.98 | 0.70 | 0.84 | 0.54 | 0.92 | 0.93 | 0.92 | 0.81 | 0.98 | 0.98 | 0.50 | 0.79 | 0.98 | 0.93 | 0.72 | 0.84 |
| X26 | 0.96 | 0.90 | 0.99 | 0.79 | 0.88 | 0.94 | 0.96 | 0.99 | 0.94 | 0.98 | 0.90 | 0.88 | 0.89 | 0.84 | 0.83 | 0.91 |
| X27 | 0.94 | 0.74 | 1.00 | 0.57 | 0.90 | 0.98 | 0.91 | 0.86 | 0.96 | 0.91 | 0.66 | 0.81 | 0.86 | 0.99 | 0.76 | 0.86 |
| X28 | 0.96 | 0.77 | 0.95 | 0.84 | 0.89 | 0.96 | 0.99 | 0.87 | 0.70 | 0.97 | 0.77 | 0.86 | 0.98 | 0.93 | 0.84 | 0.89 |
| X29 | 0.91 | 0.71 | 0.94 | 0.95 | 0.83 | 0.92 | 0.95 | 0.87 | 0.54 | 0.94 | 0.81 | 0.91 | 0.96 | 0.93 | 0.84 | 0.87 |
| X30 | 0.85 | 0.75 | 0.91 | 0.70 | 0.94 | 0.97 | 1.00 | 0.88 | 0.82 | 0.97 | 0.81 | 0.88 | 0.99 | 1.00 | 0.82 | 0.89 |
| X31 | 0.99 | 0.73 | 0.85 | 0.83 | 0.85 | 0.97 | 0.98 | 0.88 | 0.71 | 0.98 | 0.74 | 0.84 | 0.93 | 0.94 | 0.81 | 0.87 |
| X32 | 0.87 | 0.71 | 0.93 | 0.89 | 0.80 | 0.96 | 0.96 | 0.85 | 0.63 | 0.97 | 0.72 | 0.84 | 0.92 | 0.91 | 0.81 | 0.85 |
| X33 | 0.95 | 0.83 | 0.67 | 0.87 | 0.79 | 0.99 | 0.82 | 0.98 | 0.91 | 0.84 | 0.96 | 0.97 | 0.87 | 0.85 | 1.00 | 0.89 |
| X34 | 0.92 | 0.82 | 1.00 | 0.95 | 0.97 | 0.89 | 0.93 | 0.99 | 0.88 | 0.91 | 0.86 | 0.84 | 0.95 | 0.90 | 0.92 | 0.92 |
| X35 | 0.91 | 0.73 | 0.68 | 0.99 | 0.88 | 0.94 | 0.99 | 0.83 | 0.56 | 0.95 | 0.76 | 0.84 | 0.96 | 0.92 | 0.76 | 0.85 |
| X36 | 0.93 | 0.99 | 0.99 | 0.83 | 0.92 | 0.82 | 0.90 | 0.91 | 0.86 | 0.92 | 0.84 | 0.88 | 0.95 | 0.84 | 0.91 | 0.90 |
| X37 | 0.96 | 0.82 | 0.68 | 0.88 | 0.79 | 0.89 | 0.89 | 0.99 | 0.92 | 0.85 | 0.98 | 0.94 | 0.87 | 0.92 | 0.95 | 0.89 |

Table S4. Molecular docking binding energy

| Compound | AKT1  (kcal/mol) | IL-6  (kcal/mol) | TNF  (kcal/mol) |
| --- | --- | --- | --- |
| paeoniflorin | -5.9 | -8.2 | -8.5 |
| galloylpaeoniflorin | -10.9 | -7 | -10.4 |
| oleanic acid | -10.7 | -9.4 | -9.2 |
| albiflorin | -9.6 | -6.7 | -7.9 |
| benzoylpaeoniflorin | -6.5 | -5.7 | -7.6 |
| 1,2,3,4,6-o-pentagalloylglucose | -10.1 | -6.4 | -10.6 |
| pinocembrin | -5.3 | -5.1 | -5.2 |
| Isorhamnetin-3-o-nehesperidineand | -6.8 | -5.5 | -8.2 |


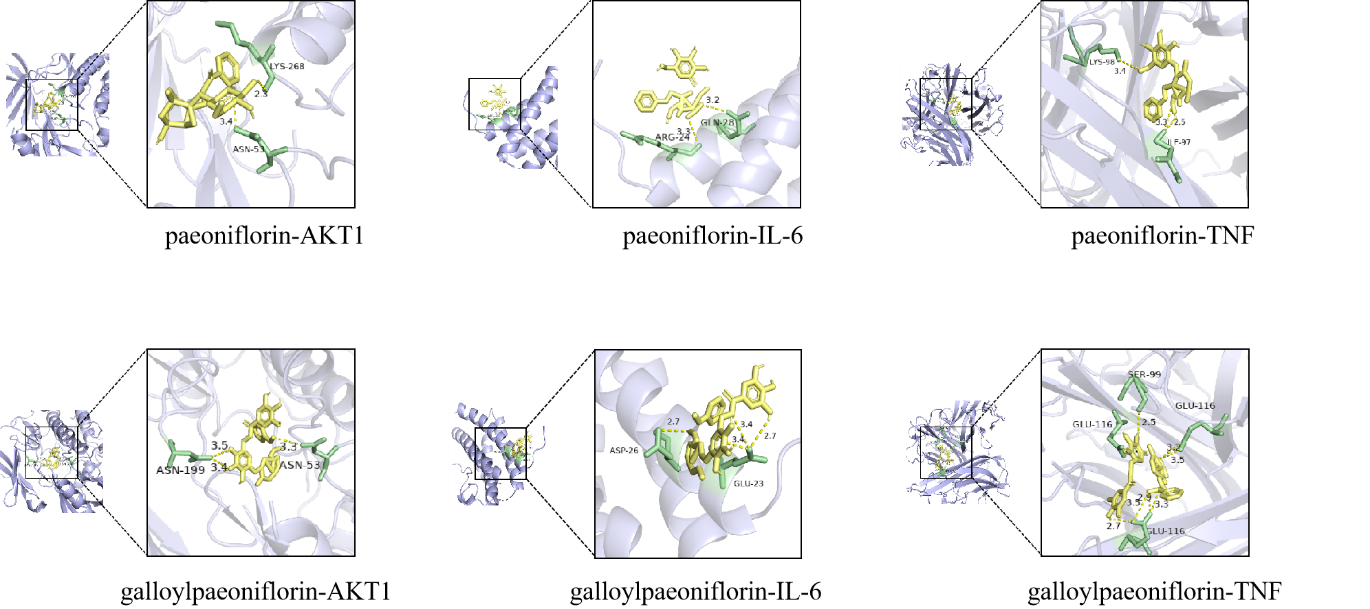


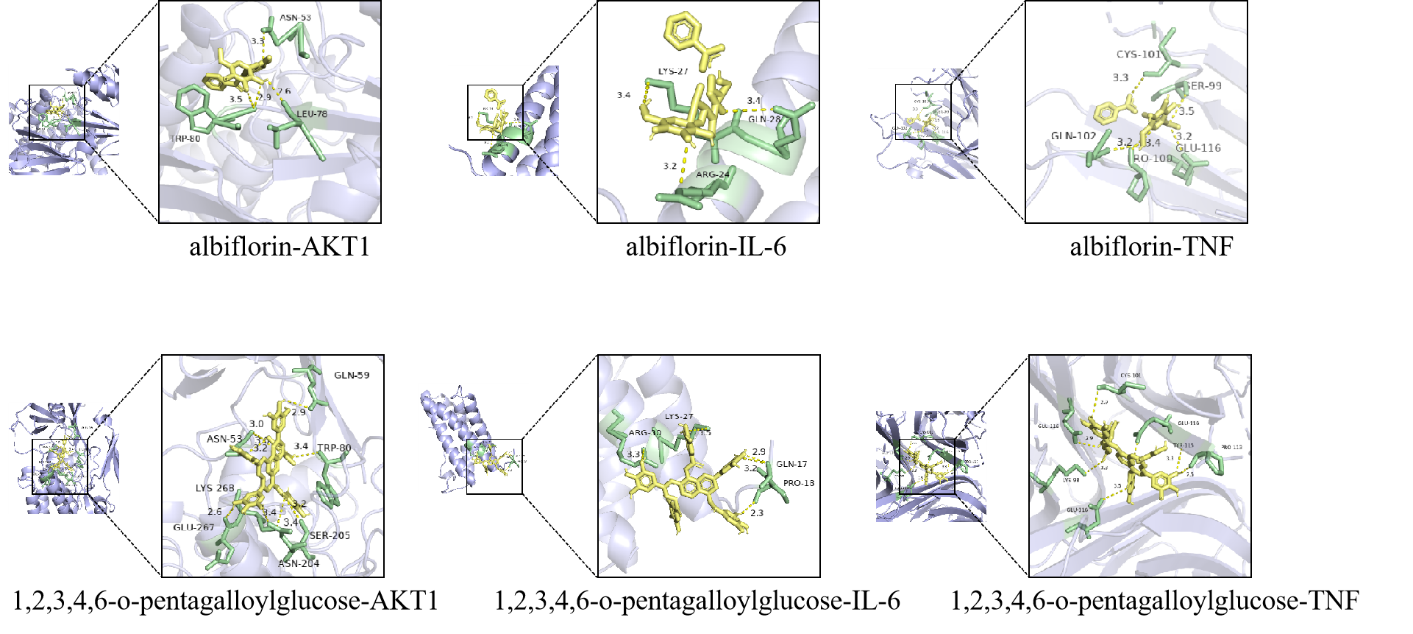


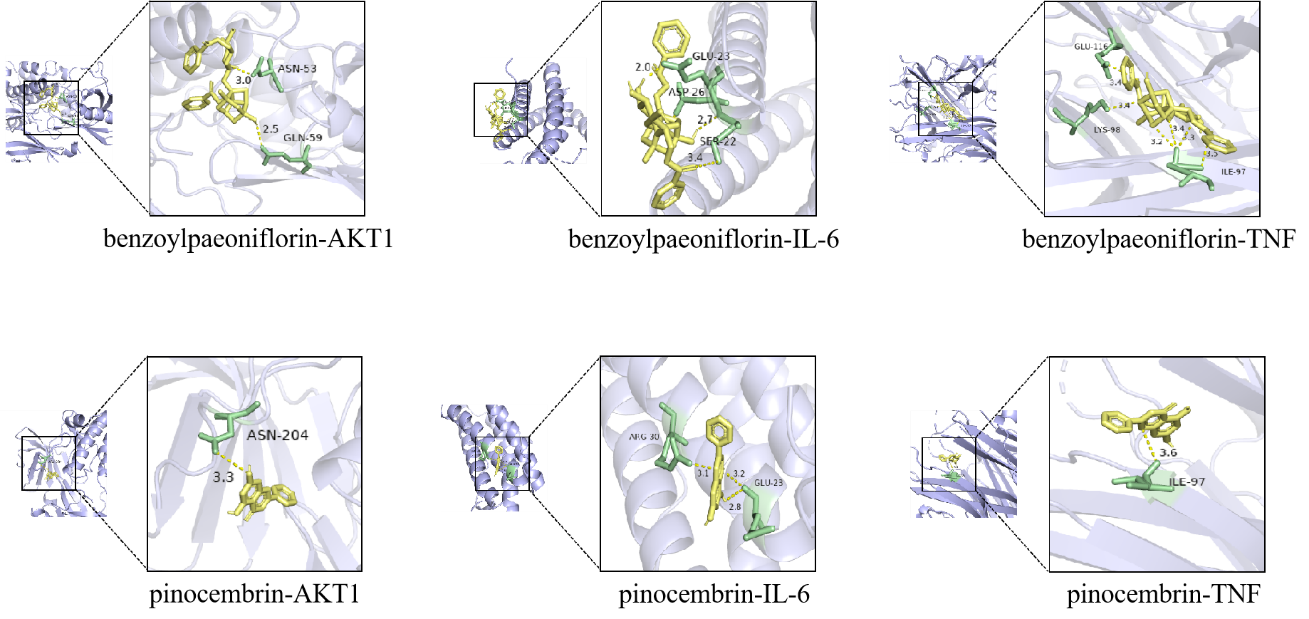


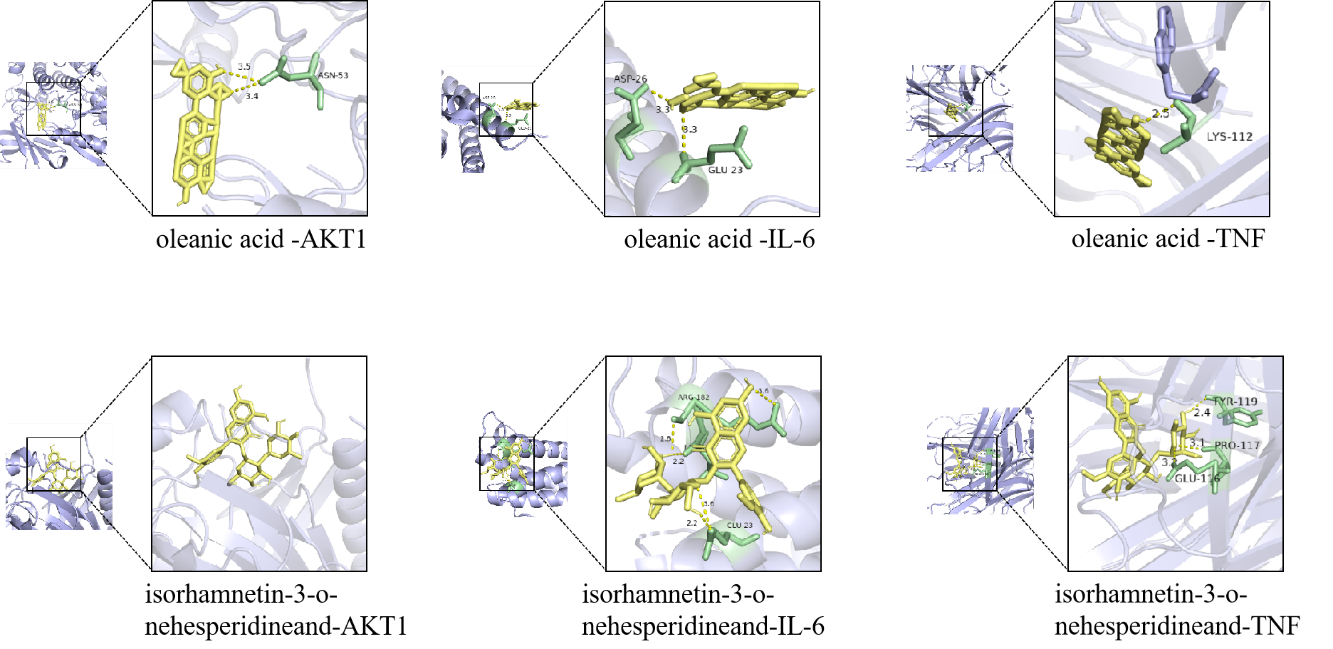


Figure S4 Molecular docking of potential targets with active conponents

Figure S5. Pearson correlation analysis of SOD, GSH and MDA
